# Strengthening alcohol consumption screening in primary care for culturally diverse populations in Latin America: Adaptation and content and face validity of the AUDIT in Guaviare, Colombia

**DOI:** 10.1371/journal.pone.0359625

**Published:** 2026-09-30

**Authors:** Andrea López-González, Adriana Buitrago-López, Nelcy Rodríguez Malagón, Nicolás Martínez Ramos, Jhon Sebastián Patiño Rueda, Deyanira Sindy Moya Chaves, Dora Yonda Suns, Carlos Gómez-Restrepo

**Affiliations:** 1 Department of Clinical Epidemiology and Biostatistics, School of Medicine, Pontificia Universidad Javeriana, Bogotá, Colombia; 2 Centro Javeriano de Oncología, Hospital Universitario San Ignacio, Bogotá, Colombia; 3 Research Line on Health Systems, Childhood, Gender, Interculturality and Tropical Diseases, Research Group on Public Health, Medical Education and Medical Professionalism, School of Medicine, Universidad de los Andes, Bogotá, Colombia; 4 Department of Language Studies, School of Communication and Language, Pontificia Universidad Javeriana, Bogotá, Colombia; 5 School of Medicine, Pontificia Universidad Javeriana, Bogotá, Colombia; School of Nursing Sao Joao de Deus, Evora University, PORTUGAL

## Abstract

**Background:**

The World Health Organization developed the Alcohol Use Disorders Identification Test, widely used to detect alcohol consumption and guide interventions in primary care. Despite its broad use, evidence of its validity in culturally diverse settings remains limited. Although Spanish versions exist, none have been culturally adapted for Guaviare, a Colombian Amazon region with multicultural communities and socioeconomic and healthcare inequities. Such inequities may intensify alcohol consumption and related consequences. Without cultural adaptation, AUDIT screening may underestimate consumption and hinder effective primary-care interventions.

**Objective:**

This study aimed to culturally adapt and assess the content and face validity of the AUDIT in both interview and self-administered formats for alcohol consumption screening among adults in Guaviare, Colombia.

**Methods:**

This study followed a descriptive qualitative approach to guide the cultural adaptation and validation of a scale. Phase 1: cultural adaptation was conducted through consensus among researchers and a panel of community experts (n = 11), using a nominal group technique to prioritize adjustments from the community’s perspective. Phase 2: Field testing involved cognitive interviews (n = 18) to assess content and face validity, as well as semantic and technical equivalence. Additional cultural refinements were made based on participants’ feedback.

**Results:**

Cultural adaptations were made to the instructions, questions, and response options. Most adjustments focused on improving comprehension of the scale and guiding respondents to answer each item accurately. In addition, modifications were implemented to make the scale more recognizable and culturally appropriate for the community.

**Conclusion:**

This study shows that the culturally adapted AUDIT for adults in Guaviare demonstrates adequate content and face validity, representing the first validated version of the scale for this population. The findings underscore the importance of context-sensitive adaptations to ensure accurate identification of alcohol consumption and to strengthen primary care interventions.

## Introduction

Alcohol consumption is increasing and represents a global public health problem due to its high morbidity and mortality, particularly in low- and middle-income countries such as Colombia [1–3]. This condition can lead to both physical and social consequences in the short and medium term [1,4]. Since 1987, the World Health Organization (WHO) has developed and validated the Alcohol Use Disorders Identification Test (AUDIT) to screen for hazardous alcohol consumption and to support interventions in primary care [5]. This scale currently has two validated Spanish versions for Latin American contexts, one from Ecuador and another from Mexico [5,6]. However, these versions have not been culturally adapted for diverse populations or for groups experiencing social and economic disadvantages in Latin America, nor do they incorporate locally relevant cultural alcohol consumption practices. Applying the scale without cultural adaptation may lead to comprehension difficulties and operational challenges during administration, ultimately compromising measurement validity [2].

The distinction between translation and cultural adaptation is critical to understanding the gap described above. Translation refers to the linguistic conversion of an instrument’s content from one language to another, aiming for lexical and grammatical equivalence. Cultural adaptation, by contrast, is a broader process that extends beyond word-for-word translation to ensure content, technical, and semantic equivalence between the original instrument and the target population, particularly when the target context differs substantially from the one in which the instrument was originally developed [7,8]. Cultural adaptation therefore involves modifying instructions, item wording, response options, and administration procedures to reflect idiomatic expressions, regionally relevant drinking practices, and the local cultural context, while ensuring that the underlying construct retains its original meaning [7,8]. This process reflects not only a methodological necessity, but also a recognition of communities’ right to actively participate in validating their own knowledge and practices, according to their own needs and context [9]. Internationally recognized guidelines, such as those proposed by the ISPOR Task Force for Translation and Cultural Adaptation [10], formalize this process into a series of systematic steps—typically involving translation, expert and community review, and cognitive assessment of respondent understanding through techniques such as cognitive interviewing [11].

Beyond linguistic differences, terminology and expressions related to alcohol consumption vary substantially across Spanish-speaking regions, shaped by the sociocultural characteristics of each area [12]. Consequently, experts have emphasized the importance of culturally adapting measurement instruments when the target population differs from that for which the scale was originally developed. This recommendation is particularly relevant in culturally diverse settings, such as Guaviare, even when a Spanish version is already available [13].

Guaviare is a culturally and ethnically diverse region located in the Colombian Amazon, home to multiple Indigenous peoples and internal migrants [14,15]. The region also faces intersecting social, environmental, and territorial challenges, including poverty, armed conflict, and limited access to healthcare and basic services [16–18]. These factors contribute to reduced quality of life and increased alcohol consumption relative to national levels [17]. The high levels of alcohol consumption in the region have likely contributed to increases in homicides, theft, domestic violence, traffic accidents, and physical injuries [19].

Primary healthcare plays a crucial role in the early detection and management of alcohol consumption in Guaviare due to the limited availability of mental health specialists. The AUDIT is one of the most strongly recommended instruments for this purpose by the WHO [5,20]. However, there is no Spanish version of the AUDIT that has been culturally adapted for the diverse population of Guaviare. Cultural adaptation plays a crucial role in addressing this gap, as it enables a clearer understanding of the instructions, questions, and response options, thereby improving the accuracy and validity of responses and facilitating the appropriate implementation of the interventions recommended by the scale [13,21]. Additionally, the cultural adaptation and validation of this scale could demonstrate a methodological approach that can be learned and replicated by researchers working in other contexts. This transferable framework creates opportunities to generate culturally appropriate measurement tools and advance knowledge in diverse communities worldwide.

This study aimed to culturally adapt and assess the content and face validity of the AUDIT in both interview and self-administered formats for alcohol consumption screening among adults in Guaviare, Colombia.

Content and face validity are two related but distinct properties commonly assessed during instrument adaptation. Content validity refers to whether the items of an adapted scale adequately represent the construct they are intended to measure, while face validity refers to whether the instrument appears, at first glance, to measure what it claims to measure from the perspective of those who administer or respond to it [22]. Together, both properties are essential when culturally adapting a scale such as the AUDIT for a new population.

## Materials and methods

### Settings and study sites

This study was conducted in two locations in Guaviare, Colombia: San José del Guaviare, the capital of the region (urban area), and the township of Charras-Boquerón (rural area). This area is home to Indigenous, Afro-descendant, and farming communities, as well as victims of the armed conflict, migrants, and others [14,15]. There are few health centers in the region due to its remote geographical location and the limited availability of financial resources and healthcare personnel. In San José del Guaviare, there is a public hospital that provides primary care and specialized medical services; however, it does not have any psychiatrists available. In contrast, in the township of Charras-Boquerón, located approximately two hours from San José del Guaviare, there is only primary care provided by a general practitioner and nursing staff.

### Study design

This was a qualitative descriptive study conducted to support the cultural adaptation and the assessment of content and face validity of the AUDIT [23,24]. The theoretical framework used in this study followed the recommendations of the International Test Commission (ITC) and the guidelines outlined in the book Measurement and the Measurement of Change proposed by Polit & Yang [8,22]. The study was divided into two consecutive phases in which qualitative data collection methods were implemented, including structured observation, the nominal group technique, and cognitive interviewing (Fig 1). The cultural adaptation was conducted on the Spanish version of the AUDIT recommended by the WHO since 2001 for use in Latin American populations. This adaptation was carried out for both the interviewer-administered and the self-administered formats (S1 Appendix) [5].

**Fig 1:**
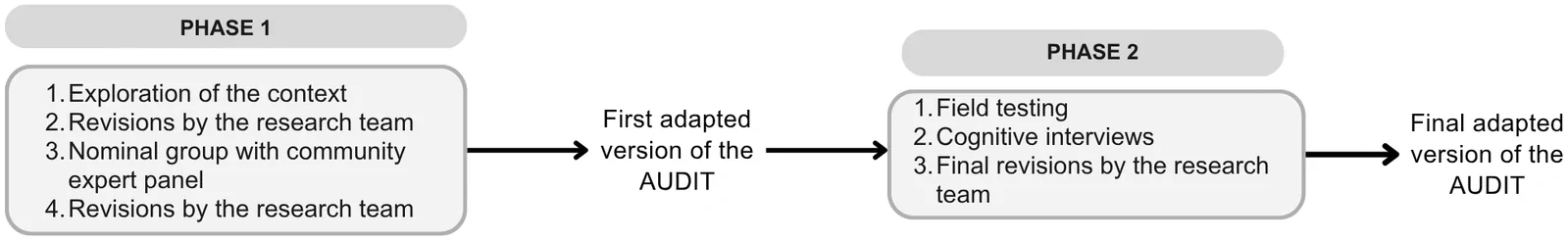
General description of the phases of the AUDIT adaptation. The cultural adaptation was divided into two consecutive phases. The first phase comprised the steps leading to the first adapted version of the AUDIT. The second phase included additional refinements that resulted in the final adapted version of the AUDIT.

### Participants

A purposive, criterion-based, non-probability sampling approach was employed to capture the sociocultural diversity of the population in Guaviare, Colombia. Participants were selected considering characteristics such as social group (settlers, peace agreement signatories, rural farmers, and Indigenous or Afro-descendant people), educational level, sex, age, and place of residence, to ensure heterogeneous representation. In the context of Guaviare, these groups reflect distinct historical and cultural backgrounds related to settlement, rural livelihoods, and ethnic identity. Individuals aged 18–65 years who were Spanish speakers with adequate comprehension and communication skills, and who were either native residents or migrants with occupational or educational integration in the department, were eligible for inclusion, provided they gave voluntary consent. Those unavailable during the data collection period were excluded.

The recruitment strategy involved gatekeeping and door-to-door approaches. Recruitment continued until all predefined sociodemographic characteristics were represented in the sample. Eleven participants were included as community experts (Phase 1), and 18 participants were selected for field testing (Phase 2). Recruitment took place from April 2, 2024, to August 5, 2024. The community experts were divided into two groups of five and six individuals, respectively—one from a rural area and the other from an urban area of Guaviare, Colombia. They participated in collaborative activities with the research team to review, discuss, and propose cultural adjustments to the AUDIT scale.

### Adaptation team

The cultural adaptation of the instrument was undertaken by an interdisciplinary team of researchers, comprising professionals with expertise in clinical epidemiology, biostatistics, alcohol consumption, public health, primary care, linguistics, and scale adaptation and validation (ALG, NMR, JSPR, DSMC, and DYS). The team included both technical personnel based in the region and experts with extensive experience working with populations in complex socio-economic contexts. Community experts (Phase 1) participated as co-creators in the adaptation process, contributing local knowledge, cultural practices, and communication styles specific to the communities of Guaviare.

### Adapted scale framework

The AUDIT is available in two formats, interview and self-administered. It was developed by the WHO for screening alcohol consumption and suggesting appropriate interventions based on the obtained score [5]. The AUDIT is freely available for research and publication use (S1 Appendix). It consists of ten items grouped into three dimensions of hazardous alcohol consumption: alcohol consumption (items 1–3), symptoms of dependence (items 4–6), and harmful alcohol consumption (items 7–10) [5,25]. Each question offers three to five ordinal response options, assigned discrete scores ranging from zero to four, allowing for the calculation of a total score between zero and 40 points. A score equal to or greater than eight indicates hazardous alcohol consumption, with a sensitivity of 90% and a specificity of 80% [5]. The WHO currently recommends the use of the Spanish version validated in Mexico for Spanish-speaking populations in Latin America [26].

### Data collection

#### Phase 1.

An initial exploration of the context of alcohol consumption was conducted through on-site structured observation (S2 Appendix) in San José del Guaviare and the township of Charras-Boquerón (Department of Guaviare, Colombia). The researcher participated in community meetings and celebrations, visited alcohol-selling establishments, and observed households where alcoholic beverages were consumed. The findings derived from these observations were shared with the research team to inform culturally appropriate adjustments aligned with the local context.

The research team met virtually to carry out the first modifications to the original version of the AUDIT (S1 Appendix). These adjustments focused on assessing the cultural relevance of each item in relation to the construct of hazardous alcohol consumption and the population of Guaviare.

Two groups of community experts were formed according to the selection criteria, and an in-person meeting was held with each group—one in San José del Guaviare and the other in Charras-Boquerón, Guaviare, Colombia. During these meetings, the nominal group technique was implemented (S3 Appendix).

Subsequently, the research team met virtually to resolve discrepancies and make further adjustments based on the results of the nominal group technique. This process led to the development of the first culturally adapted version of the AUDIT in both formats (self-administered and interview) (S4 Appendix).

#### Phase 2.

Participants in this phase completed field testing using the first adapted version of the AUDIT (S4 Appendix). For the self-administered format, the researcher provided a printed paper version of the scale, which was completed directly by the participants. In the interview format, the researcher read the instructions and questions aloud and recorded the participant’s responses on the printed form. Afterwards, the completed paper forms were entered into a digital database.

Once the scale had been completed, the researcher conducted a cognitive interview lasting approximately 30–40 minutes, using audio recording and note-taking to collect information. Cognitive interviews followed an interpretivist approach, as described by Miller et al. [27], and were analyzed through a five-step synthesis and reduction process. First, open-ended probing questions were used to explore how each participant understood and responded to each scale item (S5 Appendix); examples included “What did you understand from the instructions?”, “What did you understand by the question…?”, “What do you think you are being asked?”, “Did you have any difficulty answering the question? Why?”, “What did you understand by the answer option…?”, and “How did you arrive at that answer?” Second, responses were summarized to capture how each participant interpreted the questions, formulated their answers, and any difficulties experienced while completing the scale. Third, responses were compared across participants to identify common patterns. Fourth, findings were organized according to administration format (interview or self-administered) to identify potential differences between subgroups. Finally, conclusions were drawn regarding how participants interpreted the questions and formulated their responses, whether difficulties arose during completion, and whether differences were observed between the interview and self-administered formats. The audio recordings were transcribed verbatim, and transcription codes were applied to capture both verbal and nonverbal language.

The research team made new adjustments to the first adapted version of the AUDIT based on the results of the cognitive interviews. This process resulted in the final culturally adapted version of the AUDIT, reflecting the sociocultural context of a group of adults from Guaviare, Colombia (S6 Appendix).

### Analysis

A descriptive analysis was conducted of the participants’ sociodemographic characteristics. Errors made by participants, the number of unanswered questions, and the number of questions with multiple responses were described. In addition, the modifications made by the research team and community experts to the original AUDIT in both formats were documented.

A thematic analysis [28] of the cognitive interviews was performed to assess potential differences in interpretive patterns and response processes between participants who used the interview format and those who used the self-administered format. This analysis identified the degree of overlap between the two perspectives (researcher and participant). All cognitive interviews were transcribed and coded using a deductive, top-down thematic analysis based on a codebook containing predefined categories and subcategories (Fig 2), which were established according to the interview guide. The researcher’s field notes and participants’ responses to the AUDIT were also reviewed. The analysis focused on data reduction and the identification of broad conceptual themes. Coding was conducted using NVivo 14 software [29].

**Fig 2:**
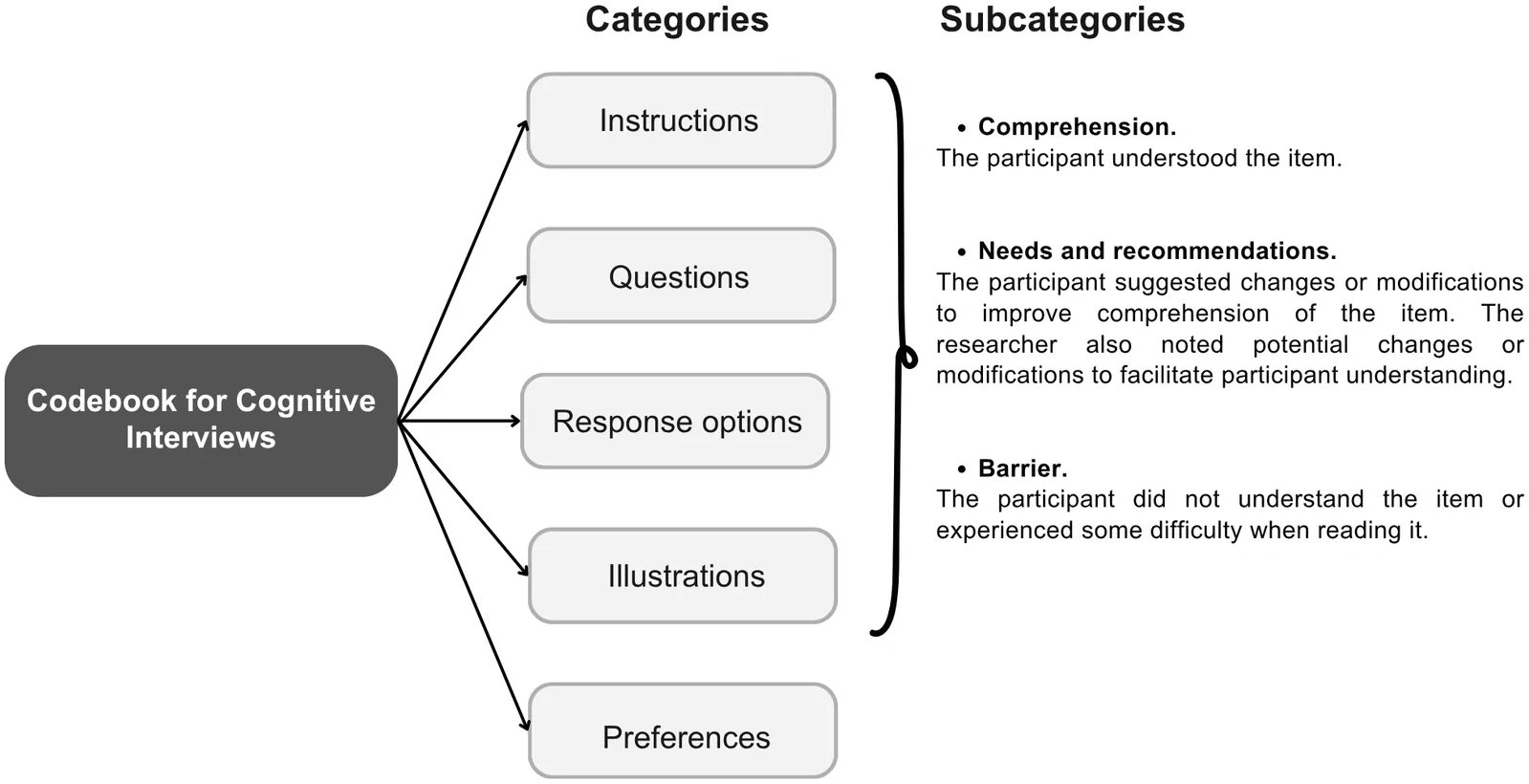
Codebook for cognitive interviews. The codebook was organized into five categories corresponding to the parts of the scale that were adapted, each classified according to three subcategories: comprehension, needs and recommendations, and barrier.

### Ethical clearance

This study was approved by the Ethics Committee of Hospital Universitario San Ignacio and Pontificia Universidad Javeriana on March 7, 2024 (Minutes No. 04/2024). All participants were adults and provided written informed consent prior to enrollment.

#### Inclusivity in global research.

The study was designed and conducted by Colombian researchers in collaboration with local stakeholders. Before data collection, the informed consent form was pilot-tested with three community members from Guaviare to ensure clarity and comprehension. As part of our ethical commitment to the Guaviare community, the study was presented to and approved by the Secretaría de Salud del Guaviare (Guaviare Department Health Secretariat), Colombia, and reviewed and endorsed by community leaders from the township of Charras-Boquerón before implementation. Community members were actively engaged throughout the cultural adaptation process through nominal group sessions and cognitive interviews, and their feedback directly informed revisions to the adapted AUDIT. To promote local capacity building, training on hazardous alcohol consumption screening was provided to healthcare personnel in Guaviare. One of the co-authors is a member of the study community. Additional information regarding the ethical, cultural, and scientific considerations specific to inclusivity in global research is provided in S7 Appendix.

## Results

### Demographic characteristics

A total of eleven individuals comprised the community expert team in Phase 1 — six from the rural area and five from urban area. Two participants were originally from San José del Guaviare, while the remaining participants were born in other Colombian regions (Cundinamarca, Boyacá, Meta, Bogotá, and Cauca) or in Venezuela. Most participants were women, over 30 years of age, and had a low educational level. Nearly half (45.5%) self-identified as belonging to a specific social group, primarily rural farmer or victim of the armed conflict (Table 1).

**Table 1:** Sociodemographic characteristics of the participants.

	Phase 1 n (%) (n = 11)	Phase 2 n (%) (n = 18)
**Sex**		
Female	7 (63.6)	8 (44.4)
Male	4 (36.4)	10 (55.6)
**Age**		
18-29	1 (9.1)	3 (16.7)
30-44	6 (54.5)	6 (33.3)
45-65	4 (36.4)	9 (50.0)
**Social group** ^**a**^		
Indigenous	0 (0.0)	1 (5.6)
Rural farmer	2 (18.2)	4 (22.2)
Victim of the armed conflict	3 (27.3)	4 (22.2)
Other	6 (54.5)	9 (50.0)
**Education level** ^**b**^		
Low	6 (54.5)	9 (50.0)
Middle	5 (45.5)	7 (38.9)
High	0 (0.0)	2 (11.1)

^a^Participants who self-identified as belonging to a social group. Rural farmer: individual living in a rural area and engaged in agricultural activities, often affected by the armed conflict and with limited economic resources [30]. *Victim of the armed conflict:* individual affected by human rights violations or breaches of international humanitarian law in the context of the Colombian armed conflict [31]. *Peace signatory*: former member of the FARC-EP who demobilized and signed the 2016 Peace Agreement with the Government of Colombia [32]. *Other:* includes participants who did not self-identify with any specific social group, as well as those who self-identified as migrant or peace signatory; these categories were combined to protect participant confidentiality given the small sample size. ^b^Educational level was categorized as follows: low (illiterate, incomplete primary, complete primary, incomplete secondary, or complete lower secondary education), middle (upper secondary or technical education), and high (undergraduate or postgraduate education). Educational level was grouped into three categories (low, middle, high) based on ISCED-derived levels within the Colombian education system [33,34] to facilitate the description of participants’ sociodemographic characteristics. Illiteracy was included within the low education level category to protect participant confidentiality, given the small sample size.

The field test (Phase 2) was conducted with 18 participants. Most participants lived in rural area (n = 10, 55.6%). Five participants were originally from San José del Guaviare, while the remaining participants were born in other Colombian regions (Cundinamarca, Meta, Bogotá, and Antioquia; n = 13, 72.2%). A total of 55.6% completed the interview-administered format, while 44.4% completed the self-administered version. Slightly more than half of participants were male and aged 45–65 years. Half of the participants self-identified as belonging to a specific social group (indigenous, rural farmer, or victim of the armed conflict), and half had a low educational level (Table 1).

### • Phase 1: Feedback from researchers

The initial adjustments to the AUDIT scale made by the research team were as follows:

- The first modification, applied to both the self-administered and interview formats, consisted of indicating the time frame (“past year”) at the beginning of each question and explicitly including the term “alcoholic beverages” to remind participants that the questions referred specifically to this type of drink.
- The following two modifications were applied only to the interview format. First, the corresponding score was added to each response option to assist healthcare personnel, researchers, or social agents in calculating the total score more quickly. Second, an explanation was included regarding how certain questions could be skipped in cases where the participant answered “never” to the first question, or when the sum of the scores for questions 2 and 3 was equal to 0 points (the participant could then proceed directly to questions 9 and 10).

During the initial meetings, several questions arose regarding the most appropriate terminology to use for certain words, such as “self-report” (other possible options: self-completed, self-filled, or self-administered), “consumption” (other possible options: use, ingest, drink, or take), and “test” (other possible options: form, survey, or format). In addition, it was determined that a definition of “alcoholic beverage” and a guide to alcoholic drinks should be included in the instructions to aid recall and reduce measurement bias. These issues were discussed with community experts using the nominal group technique.

### • Phase 1: Feedback from community experts

The main results of the nominal group technique are presented in Table 2. Five questions were asked per group. The experts shared between two and eleven ideas per question. For most questions, a consensus was reached on the responses.

**Table 2:** Results of the nominal group technique.

Questions	Most voted response – San José del Guaviare	Most voted response – Charras-Boquerón Township
How do you refer to beverages that contain alcohol?	Traditional beverages (*bebidas tradicionales*)	Alcoholic beverages (*bebidas alcohólicas*)
How do you refer to the fruit-based beverage that is fermented?	Traditional beverages (*bebidas tradicionales*)	Homemade fermented beverages (*bebidas fermentadas caseras*)
What do you do with beverages that contain alcohol?	Drink (*tomar*)	Drink (*tomar*)
How do you refer to the object used for drinking?	No consensus was reached, as it depends on the type of alcoholic beverage	Glass *(vaso*)
How do you refer to what is done with these (points to the document with questions)?	Fill out the form (*llenar el formulario*)	Fill out (*llenar*), Form (*formulario*)

The table summarizes the questions discussed during the nominal group sessions and the responses prioritized by participants in San José del Guaviare and Charras-Boquerón. Original Spanish terms are retained in parentheses when no exact English equivalent is available to preserve the intended cultural meaning.

According to the results of the nominal group technique, the following adjustments were made:

- The term “self-report” (autopase) was replaced with “self-administered” (autollenado), “register” (diligenciar) with “fill out” (llenar), “test” (cuestionario) with “form” (formulario), and “consumption” (consumir) with “drink” (tomar).
- The expression “drink alcoholic beverages” (tomar bebidas alcohólicas) was retained in all questions of the scale.
- Visual adjustments were made within the format to clarify the instructions and completion guidelines. For example, two diagrams were added to the instructions and images were included in the alcoholic beverage guide; in addition, the scoring next to each response option was removed from the self-administered version to avoid biasing participants’ responses.

The first adapted version of the AUDIT can be found in S4 Appendix.

### • Phase 2: Characteristics of the data collected using the AUDIT

Two main types of errors were observed during the completion process: selecting more than one response option or leaving an item unanswered. These errors occurred in six questions and involved six participants. They were observed in both the interview-administered and the self-administered formats. A more detailed explanation of each error is provided in Table 3.

**Table 3:** Errors made during completion of the first adapted version of the AUDIT scale (interview and self-administered formats).

Question number	Number of participants	Format	Type of error	Error explanation
1	1	Interview	More than one response	The interviewer had to remind the participant more than once of the definition of alcoholic beverages.
2	1	Self-administered	More than one response	The participant reread the question several times to recall drinking occasions.
3	2	Self-administered	More than one response	One participant reread the question several times to recall drinking occasions; another overlooked item number six.
4	1	Interview	No response	Did not understand the expression “it has been difficult for you to stop drinking alcoholic beverages.”
7	2	Self-administered	More than one response	The participants reread the question several times to recall drinking occasions.
			No response	Answered “once a year,” but was unable to select a response option.
8	2	Interview	No response	Answered “every two months,” but was unable to select a response option.
		Self-administered	No response	Answered “once a year,” but was unable to select a response option.

### • Phase 2: Results of the qualitative analysis – Cognitive interview

Below are the results of the qualitative analysis of the cognitive interviews. The summary is organized into four subsections aligned with the codebook categories. The findings were shared with the research team, who determined the necessary adjustments. Each barrier or recommendation is followed by the corresponding modification incorporated into this version of the AUDIT. Table 4 presents a comparison of the original AUDIT items and their adaptations in Phase 1 (first adapted version) and Phase 2 (final adapted version), along with the justification for each adaptation, categorized as Semantic Clarity, Comprehension and Readability, or Content Relevance; additional adjustments are provided in S6 Appendix.

**Table 4:** Comparison of the original AUDIT spanish version items and their adaptations in Phase 1 (first adapted version) and Phase 2 (final adapted version) for the self-administered and interview formats.

Item	Original version	First adapted version (phase 1)	Final adapted version (phase 2)	Justification for adaptation
1	¿Con qué frecuencia consume alguna bebida alcohólica?	En el último año,¿con qué frecuencia ha tomado alguna bebida alcohólica?	En el último año,¿con qué frecuencia ha tomado alguna bebida alcohólica?	Semantic Clarity. Comprehension and Readability
2	¿Cuántas consumiciones de bebidas alcohólicas suele realizar en un día de consumo normal?	¿Cuántas copas, vasos, cervezas, tragos o cuyas de bebidas alcohólicas suele tomar en un día de consumo normal?	¿Cuántas copas, vasos, cervezas o tragos de bebidas alcohólicas suele tomar en un día de consumo normal?	Semantic Clarity. Comprehension and Readability
3	¿Con qué frecuencia toma 6 o más bebidas alcohólicas en un solo día?	¿Con qué frecuencia toma 6 o más copas, vasos, cervezas, tragos o cuyas de bebidas alcohólicas en un solo día?	¿Con qué frecuencia toma seis (6) o más copas, vasos, cervezas o tragos de bebidas alcohólicas en un solo día?	Semantic Clarity. Comprehension and Readability. Content Relevance
4	¿Con qué frecuencia en el curso del último año ha sido incapaz de parar de beber una vez había empezado?	En el último año,¿con qué frecuencia le ha sido difícil parar de tomar bebidas alcohólicas?	En el último año,¿con qué frecuencia fue difícil parar de tomar bebidas alcohólicas después de haber empezado?	Semantic Clarity. Comprehension and Readability.
5	¿Con qué frecuencia en el curso del último año no pudo hacer lo que se esperaba de usted porque había bebido?	En el último año,¿con qué frecuencia no pudo hacer lo que se esperaba de usted porque había tomado bebidas alcohólicas?	En el último año,¿con qué frecuencia no pudo hacer lo que se esperaba de usted porque había tomado bebidas alcohólicas?	Semantic Clarity. Comprehension and Readability
6	¿Con qué frecuencia en el curso del último año ha necesitado beber en ayunas para recuperarse después de haber bebido mucho el día anterior?	En el último año,¿con qué frecuencia ha necesitado tomar alguna bebida alcohólica en ayunas para recuperarse después de haber tomado mucho el día anterior?	En el último año,¿con qué frecuencia ha necesitado tomar alguna bebida alcohólica en ayunas para recuperarse después de haber tomado mucho el día anterior?	Semantic Clarity. Comprehension and Readability
7	¿Con qué frecuencia en el curso del último año ha tenido remordimientos o sentimientos de culpa después de haber bebido?	En el último año,¿con qué frecuencia ha tenido remordimientos o sentimientos de culpa después de haber tomado bebidas alcohólicas?	En el último año,¿con qué frecuencia ha tenido remordimientos o se ha sentido culpable después de haber tomado bebidas alcohólicas?	Semantic Clarity. Comprehension and Readability
8	¿Con qué frecuencia en el curso del último año no ha podido recordar lo que sucedió la noche anterior porque había estado bebiendo?	En el último año,¿con qué frecuencia no ha podido recordar lo que sucedió la noche anterior porque había estado tomando bebidas alcohólicas?	En el último año,¿con qué frecuencia no ha podido recordar lo que sucedió la noche anterior porque había estado tomando bebidas alcohólicas?	Semantic Clarity. Comprehension and Readability
9	¿Usted o alguna otra persona ha resultado herido porque usted había bebido?	¿Usted o alguna otra persona ha resultado herido porque usted había tomado alguna bebida alcohólica?	¿Usted o alguna otra persona ha resultado herida porque usted había tomado alguna bebida alcohólica?	Semantic Clarity.
10	¿Algún familiar, amigo, médico o profesional sanitario ha mostrado preocupación por un consumo de bebidas alcohólicas o le han sugerido que deje de beber?	¿Algún familiar, amigo, médico o personal de la salud ha mostrado preocupación por su forma de tomar bebidas alcohólicas o le han sugerido que deje de tomar?	¿Algún familiar, amigo, médico o personal de la salud ha mostrado preocupación por su forma de tomar bebidas alcohólicas o le han sugerido que deje de tomar?	Semantic Clarity.

Justification for Adaptation categories: Semantic Clarity refers to modifications addressing ambiguous, unclear, or region-specific meanings of individual words or expressions within a question. Comprehension and Readability refers to modifications addressing difficulties in how a question’s structure, format, or presentation was processed or understood by participants, independent of word-level meaning. Content Relevance refers to modifications addressing whether a question’s examples or categories adequately represented the drinking practices, units of measurement, or lived experiences of the target population.

#### I) Preferences regarding the type of format (interview or self-administered).

Most participants preferred that another person ask the corresponding questions about alcohol consumption, even when they were able to read and write. This preference may be due to the perception that such questions should be asked by an expert (a physician). Another possible reason, as expressed by the participants themselves, is that some people do not like to read. However, other participants considered it preferable to answer the questions on their own.

***Charras-Boquerón Township.***

***P206:*** *“Entrevistaran… Porque pues es más fácil las preguntas pues para yo responder. O sea, pueden haber más preguntas o pueden haber menos”*

***P213:** “Lo que pasa es que les da pereza leer”*

***[Own translation]***

***P206:*** *“Interview… Because it’s easier for me to answer the questions that way. I mean, there could be more questions or there could be fewer.”*

***P213:** “What happens is that they’re too lazy to read.”*

#### II) Modification of instructions and illustrations.

Given that most participants did not know how to mark their responses to the questions, despite the presence of an explanatory note at the beginning of the scale, it was decided to include written instructions accompanied by a diagram for each question. Although most participants considered that the names and images in the visual guides for alcoholic beverages and containers were clear and contextually appropriate for the region, some offered recommendations and suggested adjustments. They recommended changing the image of “guarapo” (a traditional fermented beverage made from sugarcane juice), as this beverage is more commonly stored in a pot or gallon container. They also suggested adding other distilled alcoholic beverages consumed in the region, such as vodka and gin. In addition, participants indicated that beverages regarded as cocktails (such as “piña colada” and whisky cream) should be included. They further considered it important to add an image of a glass in the visual guide, since it is one of the most used vessels for drinking alcoholic beverages. On the other hand, participants experienced difficulties understanding the question and instructions in these visual guides, specifically because it was unclear how to mark the response immediately before the images, and the time frame referenced in the question (past year) was not specified.

#### III) Questions.

Regarding the words or expressions used in the AUDIT items—such as “injured,” “on an empty stomach,” “has shown concern,” “feelings of guilt,” and “has been unable to remember”—most participants understood them appropriately. Likewise, items 1, 5, 6, 8, 9, and 10 were the most frequently understood. This occurred regardless of whether participants lived in the township of Charras-Boquerón or in San José del Guaviare, or whether they completed the interview-administered or self-administered version of the scale.

***Charras–Boquerón Township.***

***Interviewer:*** *“en el último año,¿con qué frecuencia no pudo hacer lo que se esperaba de usted porque había tomado bebidas alcohólicas?¿Cómo entiende esta pregunta?”*

***P204:*** *“Entonces como si fuera, por ejemplo, algo que se tenía, una responsabilidad, algo que tenía pendiente, usted que no lo puede hacer por andar tomando.”*

***Interviewer:*** *“¿Algún familiar amigo médico personal de salud ha mostrado preocupación por su forma de tomar bebidas alcohólicas o le han sugerido que deje de tomar?”*

***P206:*** *“… yo creo que a ninguna mamá le gusta que su hijo tome, entonces siempre la mamá le recuerda, ay mamá es que va haber una fiesta y yo voy a ir, hijo, cuidado por allá, no se ponga por allá a tomar tanto, y usted sabe mamá que yo me comporto bien, ah bueno.”*

***[Own translation]***

***Interviewer:*** *“In the past year, how often were you unable to do what was expected of you because you had been drinking alcohol? How do you understand this question?”*

***P204:*** *“So it’s like, for example, something you had to do, a responsibility, something pending that you can’t do because you’ve been drinking.”*

***Interviewer:*** *“Has any relative, friend, doctor, or health professional expressed concern about your drinking or suggested that you stop?”*

***P206:*** *“…I think no mother likes her child to drink, so the mother always reminds them, ‘Oh, there’s going to be a party and I’m going to go, son — be careful, don’t go get so drunk,’ and you know I tell my mom I behave well, and she says, ‘ah, okay.’”*

***San José del Guaviare.***

***Interviewer:*** *“en el último año,¿con qué frecuencia ha necesitado tomar alguna bebida alcohólica en ayunas para recuperarse después de haber tomado mucho el día anterior?”*

***P217:*** *“¿No?¿El llamado guayabo? Nunca. O sea, no, no. Hay gente que sí lo hace siempre, pues toma otra cerveza para pasmar el guayabo.”*

***Interviewer:*** *con sus palabras, explique, cómo entiende esta pregunta.¿Usted o alguna otra persona ha resultado herido porque usted había tomado alguna bebida alcohólica?”*

***P201:*** *“pues porque, digamos, si tengo pareja y puedo, digamos, salir y hacerle daño a esa persona porque pues, sí salgo, de pronto, sin consentimiento, o a mi mamá que no le gusta que tome o a mis hijos. Entonces siento que eso es lastimar porque pues esas personas sufren porque, pues, obviamente, cuando uno sale a tomar algo le puede pasar, entonces está el peligro y pues porque es perjudicial.”*

***[Own translation]***

***Interviewer:*** *“In the past year, how often have you needed to drink any alcoholic beverage on an empty stomach to recover after having drunk a lot the previous day?”*

***P217:*** *“No? The so-called ‘guayabo’ [hangover]? Never. I mean, no, no. There are people who always do that — they have another beer to ease the hangover.”*

***Interviewer:*** *“In your own words, explain how you understand this question: Have you or has anyone else been injured because you had been drinking any alcoholic beverage?”*

***P201:*** *“Well, because, say, if I have a partner I could, like, go out and hurt that person because, well, I go out, maybe without consent, or my mother who doesn’t like that I drink, or my children. So I feel that that is hurting them because those people suffer, because, obviously, when one goes out to drink something things can happen — there is danger and it is harmful.”*

On the other hand, some participants had difficulties interpreting certain other words or expressions. For example, the phrase “past year” was understood as referring to the period from January of the current year to the present date, rather than to the last 12 months. Therefore, the instructions and explanation clarifying its meaning were included. Additionally, some participants had difficulty reading the number 6 in item 3 (“How often do you have six or more...?”), which led to misinterpretation of this item. This issue occurred mainly among those who completed the self-administered version. Consequently, it was decided to write out the number “six” in words and highlight it in bold.

The word “cuya”—a term referring to a traditional vessel used for drinking alcoholic beverages in Indigenous communities—had been introduced by the research team during Phase 1 of the study as a potential cultural adaptation. However, when included in the questions and response options (items 2 and 3) (“…cups, glasses, beers, drinks, or cuyas…”), it caused confusion among participants during reading. Therefore, this word was removed. Furthermore, some participants had difficulty interpreting the expression “feelings of guilt” in item 7. This problem also appeared more frequently in the self-administered format. Consequently, this expression was replaced with “have you felt guilty,” which participants found clearer during the interviews.

***Charras–Boquerón Township.***

***Interviewer:*** *“¿Qué significa sentimientos de culpa?”*

***P205:*** *“Sentimientos de culpa es ponerme a pensar cosas, pongamos en mí, que tengo problemas sentimentales,¿ya? Entonces, si yo pienso que esos problemas sentimentales los voy a arreglar tomando, me dedicaría a tomar.”*

***[Own translation]***

***Interviewer:*** *“What does feelings of guilt mean?”*

***P205:*** *“Feelings of guilt means thinking about things—let’s say, in my case, I have relationship problems, right? So, if I think that I’m going to solve those problems by drinking, I would just dedicate myself to drinking.”*

Most participants had difficulties reading and understanding item 4 (“How often have you found it difficult to stop...?”). Some needed to read it several times, while others interpreted it in different ways. For instance, they thought it referred to permanently quitting alcohol consumption or to situations in which one must stop drinking due to obligation (such as illness or high-risk occupations). In most cases, after rereading the question or when it was read aloud in a different way (during the interview format), participants understood it correctly and adjusted their responses. Therefore, the wording of this question to make it easier to read and to improve comprehension was modified.

***Charras–Boquerón Township.***

***Interviewer:*** *[Se repite pregunta nuevamente y se hace énfasis en donde ha tenido dificultades en la interpretación] “Con qué frecuencia le ha sido difícil parar de tomar bebidas alcohólicas?”*

***P203:*** *“Bueno, parar es cuando uno está tomando, pues a mí nunca me ha hecho difícil. Nunca. Nunca, cuando se digan no más, eso no más. Ahora sí la entendí bien.”*

***[Own translation]***

***Interviewer:*** *[The question was repeated, emphasizing the part that had caused difficulty in interpretation] “How often has it been difficult for you to stop drinking alcoholic beverages?”*

***P203:*** *“Well, stopping means when one is drinking, right? For me, it has never been difficult. Never. Never—when I say that’s it, that’s it. Now I understood it well.”*

***San José del Guaviare.***

***Interviewer:*** *“En el último año,¿con qué frecuencia le ha sido difícil parar de tomar bebidas alcohólicas?”*

***P212:*** *“Pues, siempre trato de bajarle,¿sí me entiendes? Al consumo, entonces, pensé en eso, como es, las veces que quiero dejar de bajarle, porque no es bueno el alcohol, obviamente.”*

***[Own translation]***

***Interviewer:*** *“In the past year, how often has it been difficult for you to stop drinking alcoholic beverages?”*

***P212:*** *“Well, I always try to cut back, you know? On my drinking. So I thought about that—about the times I’ve wanted to cut back, because alcohol isn’t good, obviously.”*

Although the visual guides and the definitions of alcoholic beverages helped participants recall occasions when they had consumed such drinks at the beginning of the form, some forgot these instructions when answering the first three questions. Some individuals continued to think only about episodes of intoxication rather than more frequent consumption involving smaller amounts of alcohol, while others considered only distilled alcoholic beverages.

For this reason, the definition of an alcoholic beverage at the beginning of the form and immediately before the first three questions was included. In addition, the instructions emphasized to the interviewer (in the interview-administered format) the importance of continually reminding participants of this definition and of the alcoholic beverages they had identified in the visual guide.

***Charras–Boquerón Township.***

***Interviewer:*** *“¿Pensaste en el guarapo?”*

***P204:*** *“No, en la cerveza como tal. En la cerveza. Sí, porque el guarapo no lo considero como alcohol, pero sí, claro, eso lo emborracha a uno.”*

***Interviewer:*** *“¿Y cada cuánto toma guarapo?”*

***P204:*** *“Mmm, pues, eso sí se toma todos los días en la casa. Se bate todos los días y se toma”*

***[Own translation]***

***Interviewer:*** *“Were you thinking about guarapo (a traditional homemade fermented drink made from sugarcane)?”*

***P204:*** *“No, about beer itself. Beer. Yes, because I don’t really consider guarapo as alcohol, but of course, it does make you drunk.”*

***Interviewer:*** *“And how often do you drink guarapo?”*

***P204:*** *“Mmm, well, we drink it every day at home. It’s prepared every day and we drink it.”*

***San José del Guaviare.***

***Interviewer:*** *“¿Qué significa un día de consumo normal?”*

***P217:*** *“que todos los días se emborracha”*

***[Own translation]***

***Interviewer:*** *“What does a normal drinking day mean?”*

***P217:*** *“That you get drunk every day.”*

#### IV) Response options.

Regarding the response options, confusion arose with the expressions “once or less per month” and “less than once a month.” Although some participants understood both expressions correctly, several had difficulties when responding, and the interviewer had to provide a more detailed explanation of what that response option meant. Considering this difficulty in comprehension, an explanation of its meaning in the instructions was included.

***Charras–Boquerón Township.***

***P13:*** *“Una vez en el año también. Una vez al año no hace daño, entonces. Lo digo porque, si a veces por borracheras he tenido más accidentes en una moto. Pero falta 1 vez al año”.*

***Interviewer:*** *“¿Qué significa para usted una o menos veces al mes?”*

***P206:*** *“que tomaría una sola vez en el mes, o hay veces se pasa el mes y no toma.”*

***[Own translation]***

***P13:*** *“Once a year too. Once a while doesn’t hurt, you know? I say that because sometimes, when I’ve been drunk, I’ve had more motorcycle accidents. But just once a year.”*

***Interviewer:*** *“What do you understand by ‘once or less than once a month’?”*

***P206:*** *“That I would drink only once in the month, or sometimes a whole month goes by and I don’t drink.”*

***San José del Guaviare.***

***Interviewer:*** *“¿Qué significa para usted una o menos veces al mes?”*

***P215: “*** *que, por decir algo, este mes puedo tomar una o dos veces, el siguiente mes no puedo tomar como así sucesivamente puede que sí o a que no”*

***[Own translation]***

***Interviewer:*** *“What does ‘once or less than once a month’ mean to you?”*

***P215:*** *“That, let’s say, this month I might drink once or twice, the next month I might not drink — and so on, sometimes yes, sometimes no.”*

## Discussion

This study represents the first cultural adaptation of the AUDIT in a population facing economic and social challenges, such as that of Guaviare, Colombia. To date, only one adaptation of this scale (interview format) has been conducted in the country, specifically among a university population in Bogotá [35]. There are notable differences between the adaptation proposed by Riveros et al. and the present study. For instance, the previous adaptation neither defined “alcoholic beverage” nor modified the term “consumption” in AUDIT’s item 1 [35]. These differences are likely attributable to the characteristics of the study population and the absence of qualitative methodologies during the adaptation phase—such as interviews or focus groups to explore community perceptions and language comprehension. In contrast, we implemented several cultural adjustments and added instructions and visual guides of alcoholic beverages and containers according to the sociocultural context. These modifications aimed to reduce measurement bias and facilitate participants’ ability to recall and accurately comprehend each question and response option.

Although the WHO AUDIT manual suggests that this scale can be used in any cultural context, the social dynamics surrounding alcohol consumption in each community—particularly in those with disadvantaged socioeconomic conditions—lead to variations in language and in the definitions of alcoholic beverages. Therefore, the absence of an adaptation for such communities may result in measurement errors and have implications for both clinical practice and research processes [5,36]. In this regard, having this new version of the AUDIT adapted for adults in Guaviare represents an opportunity to improve the early detection and timely treatment of this issue in the region. Likewise, cultural adaptations related to alcohol consumption are particularly important in low- and middle-income countries, as they allow for more valid screening in settings where this problem is increasingly [36].

Although the ideal administration of the AUDIT is based on the use of a standard drink guide, in this study a visual guide depicting the different types of alcoholic beverages commonly consumed in the region, as well as a guide to the containers used for their consumption, was included in order to facilitate participants’ recall and understanding of the questions [36,37]. This decision was made for several reasons. The main one is that, in Colombia, there is currently no official definition of a standard drink, and it is not advisable to use standard drink guides from other countries, given that the Guaviare department is multicultural and it is common for individuals to consume traditional beverages that are homemade, produced through non-standardized methods, and have variable or unknown alcohol content. Furthermore, the community uses various types of containers to drink these beverages, making it difficult to objectively determine the milliliters consumed per person. In addition, terminology such as “grams of alcohol” or “standard alcoholic drink” is unfamiliar to the population of Guaviare and could cause confusion when responding to the questionnaire items.

Notably, the cultural adaptation process revealed a form of cultural invisibility surrounding alcohol consumption: traditional fermented beverages such as “guarapo” were not initially perceived by participants as alcoholic beverages, despite being one of the most frequently consumed alcoholic beverages in rural regions of Colombia [38]. This finding suggests that culturally embedded drinking practices may remain unrecognized as alcohol consumption, potentially compromising the validity of alcohol consumption measures in this population. In response to this finding, several cultural adaptations were made to the instrument, including the incorporation of a definition of alcoholic beverages that explicitly included fermented beverages, a visual guide illustrating these beverages, and repeated reminders of this definition throughout the instrument. These modifications were intended to reduce measurement bias by helping participants recognize fermented beverages as alcoholic beverages and recall occasions on which they had consumed alcohol. Nevertheless, it is important to acknowledge that a measurement of bias due to individual underreporting may persist—a phenomenon widely documented in the literature and frequently observed when assessing alcohol consumption [39].

Following the linguistic and cultural adaptation of the AUDIT scale, all items were found to be relevant and comprehensive with respect to the construct of hazardous alcohol consumption among adults in Guaviare. On the other hand, the type of format or method of administration of the scale (interview or self-administered) is a subjective decision that depends on each participant. In other words, based on the results of Phase 2 of this study, it is not possible to recommend using only the interview or the self-administered format in clinical or research contexts. Several studies have suggested that allowing participants to choose the format may increase the validity of their responses, particularly in populations with sociocultural diversity or low educational levels [40,41]. For this reason, a question regarding participants’ format preference was included in the instructions.

## Limitations

The sample size was determined using a purposive, criterion-based approach aimed at ensuring heterogeneous representation across relevant sociodemographic characteristics, rather than information saturation or interpretative redundancy, which were not formally assessed; it is therefore possible that additional participants could have contributed further insights not captured in the current analysis. Because alcohol consumption is a sensitive topic, participants in the cognitive interviews may have been reluctant to openly express difficulties in understanding certain items, potentially limiting the depth of the qualitative feedback obtained. This reluctance may also reflect the influence of social desirability, a well-documented source of bias in self-reported alcohol consumption. In addition, because all participants were Spanish-speaking, the cultural adaptation findings may not be generalizable to populations who do not speak Spanish. Finally, this study assessed only the content and face validity of the culturally adapted AUDIT. Other psychometric properties, including reliability and construct validity, are currently being evaluated and will be reported in a subsequent publication.

## Conclusion

This study presents the first culturally adapted version of the AUDIT for adults in the Colombian Amazon, addressing a critical gap in validated screening instruments for populations that have historically had limited access to context-appropriate assessment tools. This process yielded a Spanish-language version of the AUDIT with robust evidence of content and face validity, tailored to the cultural realities of Guaviare. These findings also underscore the importance of cultural adaptation across different contexts, as alcohol-related practices vary substantially between populations; even traditional beverages such as “guarapo” can become normalized to the point of not being recognized as alcoholic, requiring careful adjustments to improve the accuracy of alcohol consumption measurement. This methodological approach offers a transferable framework for adapting and validating screening instruments in other diverse or resource-constrained settings, contributing to global efforts to strengthen early detection of alcohol consumption and improve public health strategies worldwide.

## Supporting information

S1 Appendix Original AUDIT versions (interviewer and self-administered formats). (DOCX)

S2 Appendix Structured observation guides. (DOCX)

S3 Appendix Nominal group technique guide. (DOCX)

S4 Appendix First adapted AUDIT versions (interviewer and self-administered formats). (DOCX)

S5 Appendix Cognitive interview guide. (DOCX)

S6 Appendix Final adapted AUDIT versions (interviewer and self-administered formats). (PDF)

S7 Appendix Inclusivity in global research questionnaire. (DOCX)

S8 Appendix Dataset. (XLSX)
