# Supplementary material for: Strengthening alcohol consumption screening in primary care for culturally diverse populations in Latin America: Adaptation and content and face validity of the AUDIT in Guaviare, Colombia
Source: PLoS One. 2026 Sep 30;21(9):e0359625. doi: 10.1371/journal.pone.0359625 (PMC13626350; doi:10.1371/journal.pone.0359625)
Supplement: Supplementary file 1 [file pone.0359625.s006.pdf]

This appendix presents the first adapted Spanish version of the Alcohol Use Disorders Identification Test (AUDIT), resulting from the cultural adaptation process conducted in Guaviare, Colombia. Both the interviewer and self-administered formats are included. This version constitutes one of the study outcomes and is therefore presented in the language in which it was culturally adapted. An English version is not provided because it was not developed or evaluated as part of this study. For reference, the original English version of the AUDIT is provided in S1 Appendix.

Centro NIHR de Investigación en Salud Global

Test de Identificación de Trastornos por Consumo de Alcohol (AUDIT)

Adaptación realizada por:

Andrea López González
Danuby Adriana Buitrago López
Nelcy Rodríguez Malagón
Sebastián Patiño Rueda
Nicolás Martínes Ramos
Deyanira Sindy Moya Chaves

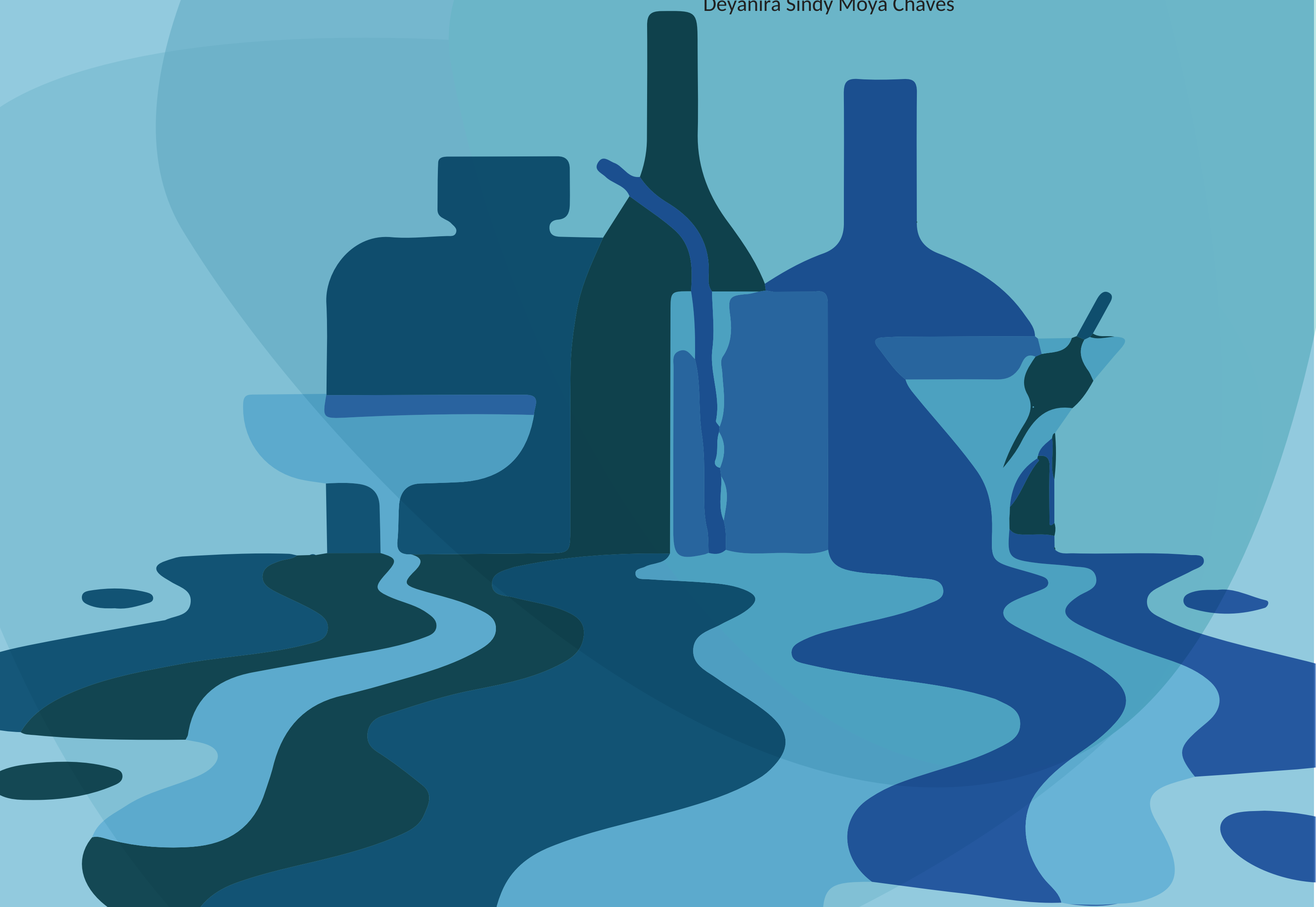

Published with permission from Pontificia Universidad Javeriana under a CC BY 4.0 license.
Original copyright © 2024.

Versión autollenado

PARTE I

Instrucciones para persona encargada: A continuación, va a encontrar un formulario con preguntas sobre cómo las personas toman bebidas alcohólicas para saber si esto está afectando su salud. Este formulario tiene dos versiones: una versión de autollenado que solo debe ser llenada por el participante y una versión de entrevista que debe ser llenada junto con un personal de salud o agente social o investigador.

El formato que actualmente está leyendo es la versión autollenado. Siga las siguientes instrucciones:

MOMENTO 1

Tiene el formato de autollenado vacío

1. Lea lo siguiente al participante:
"las siguientes preguntas son sobre cómo usted toma bebidas alcohólicas. Necesitamos saber si esto está afectando su salud".

2. Haga la siguiente pregunta:
"¿Aprendió a leer y escribir?".

- Sí sabe leer y escribir el participante.
  3. **Haga la siguiente pregunta** "¿Prefiere llenar el formulario solo o que una persona le ayude?".
     - Si prefiere llenar el formulario solo.
       4. Entregue el formulario versión autollenado (parte II) al participante
          Permita que responda las preguntas en privado.
     - Si prefiere que una persona le ayude.
       3. Entregue el formulario versión entrevista al participante.
          Lleve al participante donde se encuentra la persona que realizará la entrevista.
- No sabe leer ni escribir el participante
  3. Entregue el formulario versión entrevista al participante.
     Lleve al participante donde se encuentra la persona que realizará la entrevista.

MOMENTO 2

Tiene el formato de autollenado completo

1. Verifique que:
   - ✔ El formulario está lleno en su totalidad.
     - ✘ Si hace falta alguna respuesta, solicítele al participante que la complete.
   - ✔ Cada pregunta solo tenga una respuesta.
     - ✘ Si alguna pregunta tiene más de una respuesta, indíquele al participante que escoja solo una opción, la que mejor responda a la pregunta.
2. Encuentre los puntos y escriba el valor en el círculo azul a la derecha de cada pregunta.
3. Sume los puntos de todas las preguntas.
4. Escriba el resultado de la suma en el último cuadro en blanco donde dice "total de puntos".

Adaptación realizada por: Andrea López González, Danuby Adriana Buitrago López, Nelcy Rodríguez Malagón, Sebastián Patiño Rueda, Nicolás Martínes Ramos y Deyanira Sindy Moya Chaves

Fuente adaptada de: Babor TF, Higgins-Biddle JC, Saunders JB, Monteiro MG. AUDIT formulario de identificación de los transtornos debidos al consumo de alcohol [Internet]. s debidos al consumo de alcohol [Internet]. Organización Mundial de la Salud. Ginebra, Suiza; 2001 [citado el 14 de febrero de 2023]. Disponible en: https://www.paho.org/hq/dmdocuments/2009/AUDIT_spa.pdf. q/dmdocuments/2009/AUDIT_spa.pdf.

Published with permission from Pontificia Universidad Javeriana under a CC BY 4.0 license. Original copyright © 2024.

Versión autollenado

PARTE II

Responda las siguientes 10 preguntas sobre cómo usted toma bebidas alcohólicas para saber si esto está afectando su salud. Le tomará 10 minutos aproximadamente. Encontrará 7 pasos y 2 cuadros con imágenes para ayudarle a responder las preguntas.

Los cículos de color azul deben ser llenados por la persona encargada

Los cículos de color blanco deben ser llenados por el participante

Instrucciones para el participante:

1. Marque con una X la opción que mejor responde a su caso.
2. No hay respuestas buenas o malas.
3. Responda con sinceridad.
4. Puede pedir más de un formulario en caso de que se equivoque o desee volver a empezar.

PASO 1

¡RECUERDE! Una **bebida alcohólica** hace referencia a cualquier bebida comercial, casera o fermentada que contiene alcohol. Piense las veces que ha tomado alguna de estas bebidas, incluso para bajar la sed o para refrescarse. Recuerde cuando toma estas bebidas durante la semana o los fines de semana. Cuando decimos **"en el último año"**, estamos hablando del tiempo que ha pasado desde hace 12 meses hasta hoy.

Escriba una (X) en el círculo debajo de la imagen de la bebida alcohólica comercial, casera o fermentada que haya tomado en el último año. Puede marcar más de una opción.

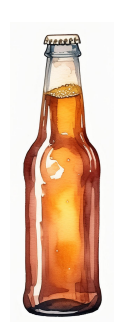
○ Cerveza, pola o coronita en botella

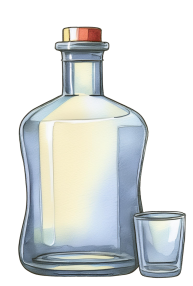
○ Aguardiente o guaro

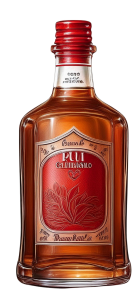
○ Ron

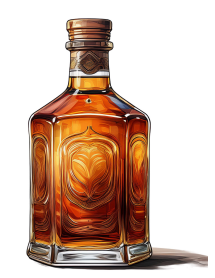
○ Whiskey

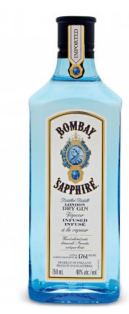

○ Ginebra

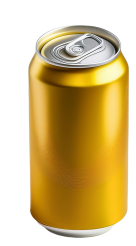
○ Cerveza, pola o coronita en lata

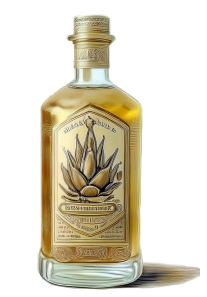
○ Tequila

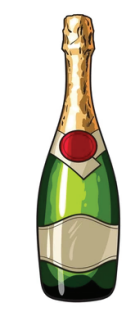
○ Champaña

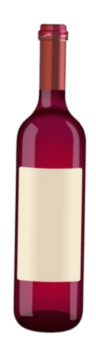
○ Vino

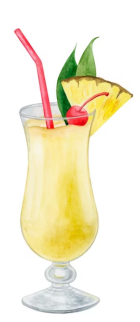
○ Cocteles piña colada

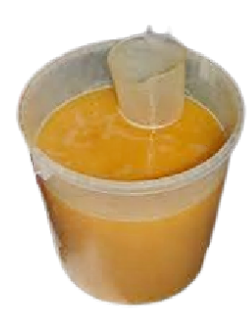
○ Guarapo

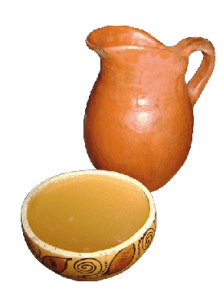
○ Chicha

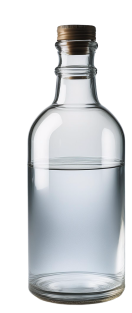
○ Viche

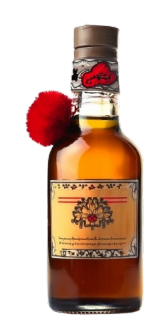
○ Chirrinchi

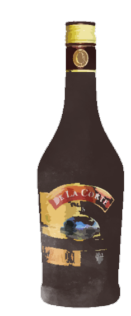
○ Crema de Whiskey

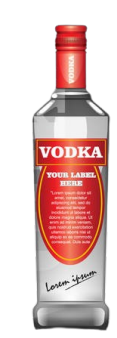

○ Vodka

PASO 2

¡RECUERDE! Una **bebida alcohólica** hace referencia a cualquier bebida comercial, casera o fermentada que contiene alcohol. Piense las veces que ha tomado alguna de estas bebidas, incluso para bajar la sed o para refrescarse. Recuerde cuando toma estas bebidas durante la semana o los fines de semana. Recuerde los ejemplos de bebidas alcohólicas en el paso 1. Responda la siguiente pregunta.

1. En el último año, ¿con qué frecuencia ha tomado alguna bebida alcohólica? Escriba una (X) sobre su respuesta

Nunca	Una o menos veces al mes	2 o 4 veces al mes	2 o 3 veces a la semana	4 o más veces a la semana
○	○	○	○	○

Adaptación realizada por: Andrea López González, Danuby Adriana Buitrago López, Nelcy Rodríguez Malagón, Sebastián Patiño Rueda, Nicolás Martínes Ramos y Deyanira Sindy Moya Chaves
Fuente adaptada de: Babor TF, Higgins-Biddle JC, Saunders JB, Monteiro MG. AUDIT formulario de identificación de los transtornos debidos al consumo de alcohol [Internet].
Organización Mundial de la Salud. Ginebra, Suiza; 2001 [citado el 14 de febrero de 2023]. Disponible en: https://www.paho.org/hq/dmdocuments/2009/AUDIT_spa.pdf.

Published with permission from Pontificia Universidad Javeriana under a CC BY 4.0 license. Original copyright © 2024.

Versión autollenado

PASO 3

Escriba una (X) en el círculo debajo de las imágenes de los recipientes que use para tomar bebidas alcohólicas comerciales, caseras o fermentadas. Puede marcar más de una opción.

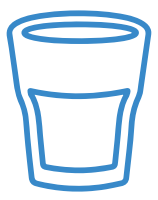
Vaso pequeño

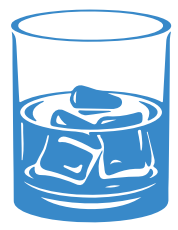
Vaso mediano

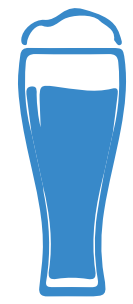
Vaso grande

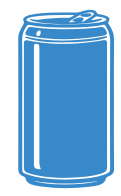
Lata

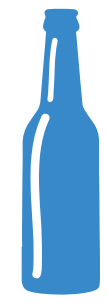
Botella

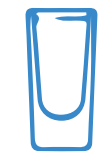
Copa delgada

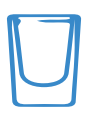
Copa pequeña

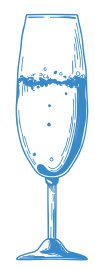
Copa larga

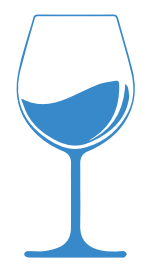
Copa Grande

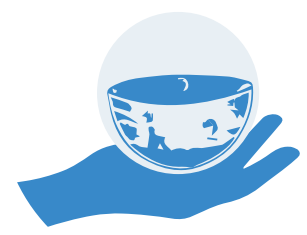
Totuma grande

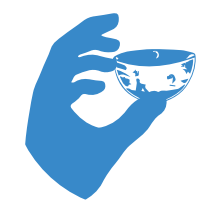
Totuma pequeña

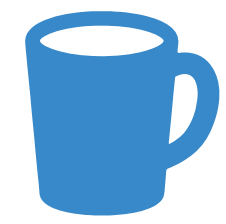
Pocillo

PASO 4

¡RECUERDE! Una bebida alcohólica hace referencia a cualquier bebida comercial, casera o fermentada que contiene alcohol. Piense las veces que ha tomado alguna de estas bebidas, incluso para bajar la sed o para refrescarse. Recuerde cuando toma estas bebidas durante la semana o los fines de semana. Recuerde los ejemplos de bebidas alcohólicas en el paso 1 y de recipientes en el paso 3. Responda la siguiente pregunta.

2. ¿Cuántas copas, vasos, cervezas o tragos de bebidas alcohólicas suele tomar en un día de consumo normal? Escriba una (X) sobre su respuesta

1 o 2 copas, vasos, cervezas o tragos	3 o 4 copas, vasos, cervezas o tragos	5 o 6 copas, vasos, cervezas o tragos	7, 8 o 9 copas, vasos, cervezas o tragos	10 o más copas, vasos, cervezas o tragos
○	○	○	○	○

PASO 5

Las siguientes preguntas tienen estas opciones de respuesta:

- Nunca que significa ni una sola vez en el último año.
- Menos de una vez al mes que significa cada 2 meses, cada 3 meses, cada 4 meses, cada 5 meses, cada 6 meses, cada 7 meses, cada 8 meses, cada 9 meses, cada 10 meses, cada 11 meses, cada 12 meses.
- Mensualmente que significa cada mes.
- Semanalmente que significa cada semana.
- A diario o casi a diario que significa todos los días o casi todos los días.

Adaptación realizada por: Andrea López González, Danuby Adriana Buitrago López, Nelcy Rodríguez Malagón, Sebastián Patiño Rueda, Nicolás Martínes Ramos y Deyanira Sindy Moya Chaves
Fuente adaptada de: Babor TF, Higgins-Biddle JC, Saunders JB, Monteiro MG. AUDIT formulario de identificación de los transtornos debidos al consumo de alcohol [Internet]. Organización Mundial de la Salud. Ginebra, Suiza; 2001 [citado el 14 de febrero de 2023]. Disponible en: https://www.paho.org/hq/dmdocuments/2009/AUDIT_spa.pdf.

Published with permission from Pontificia Universidad Javeriana under a CC BY 4.0 license. Original copyright © 2024.

Versión autollenado

3. ¿Con qué frecuencia toma **seis (6) o más** copas, vasos, cervezas o tragos de bebidas alcohólicas en un solo día?
Recuerde las bebidas que usted marcó. Escriba una Ⓧ sobre su respuesta

Nunca	Menos de una vez al mes	Mensualmente	Semanalmente	A diario o casi a diario
◯	◯	◯	◯	◯

PASO 6

Cuando decimos "en el último año", estamos hablando del tiempo que ha pasado desde hace 12 meses hasta hoy.
Responda las siguientes preguntas.

4. En el último año, ¿con qué frecuencia fue difícil parar de tomar bebidas alcohólicas después de haber empezado?
Escriba una Ⓧ sobre su respuesta

Nunca	Menos de una vez al mes	Mensualmente	Semanalmente	A diario o casi a diario
◯	◯	◯	◯	◯

5. En el último año, ¿con qué frecuencia no pudo hacer lo que se esperaba de usted porque había tomado bebidas alcohólicas?
Escriba una Ⓧ sobre su respuesta

Nunca	Menos de una vez al mes	Mensualmente	Semanalmente	A diario o casi a diario
◯	◯	◯	◯	◯

6. En el último año, ¿con qué frecuencia ha necesitado tomar alguna bebida alcohólica en ayunas para recuperarse después de haber tomado mucho el día anterior?
Escriba una Ⓧ sobre su respuesta

Nunca	Menos de una vez al mes	Mensualmente	Semanalmente	A diario o casi a diario
◯	◯	◯	◯	◯

7. En el último año, ¿con qué frecuencia ha tenido remordimientos o se ha sentido culpable después de haber tomado bebidas alcohólicas?
Escriba una Ⓧ sobre su respuesta

Nunca	Menos de una vez al mes	Mensualmente	Semanalmente	A diario o casi a diario
◯	◯	◯	◯	◯

Published with permission from Pontificia Universidad Javeriana under a CC BY 4.0 license. Original copyright © 2024.

Versión autollenado

8. En el último año, ¿con qué frecuencia no ha podido recordar lo que sucedió la noche anterior porque había estado tomando bebidas alcohólicas?
Escriba una Ⓧ sobre su respuesta

Nunca	Menos de una vez al mes	Mensualmente	Semanalmente	A diario o casi a diario
○	○	○	○	○

PASO 7

Cuando decimos "en el último año", estamos hablando del tiempo que ha pasado desde hace 12 meses hasta hoy.
Responda las siguientes preguntas.

9. ¿Usted o alguna otra persona ha resultado herida porque usted había tomado alguna bebida alcohólica?
Escriba una Ⓧ sobre su respuesta

No	Sí, pero no en el curso del último año	Sí, en el último año
○	○	○

10. ¿Algún familiar, amigo, médico o personal de la salud ha mostrado preocupación por su forma de tomar bebidas alcohólicas o le han sugerido que deje de tomar?
Escriba una Ⓧ sobre su respuesta

No	Sí, pero no en el curso del último año	Sí, en el último año
○	○	○

Total de puntos

Muchas gracias por responder todas las preguntas.
Entregue este formulario a la persona encargada.

Adaptación realizada por: Andrea López González, Danuby Adriana Buitrago López, Nelcy Rodríguez Malagón, Sebastián Patiño Rueda, Nicolás Martínes Ramos y Deyanira Sindy Moya Chaves
Fuente adaptada de: Babor TF, Higgins-Biddle JC, Saunders JB, Monteiro MG. AUDIT formulario de identificación de los transtornos debidos al consumo de alcohol [Internet]. Organización Mundial de la Salud. Ginebra, Suiza; 2001 [citado el 14 de febrero de 2023]. Disponible en: https://www.paho.org/hq/dmdocuments/2009/AUDIT_spa.pdf.

Published with permission from Pontificia Universidad Javeriana under a CC BY 4.0 license. Original copyright © 2024.

Centro NIHR de Investigación en Salud Global

Guía de puntuación versión autollenado

Instrucciones para la persona encargada:

1. Utilice esta guía para calcular el puntaje total.
2. Revise la respuessta que marcó el participante en el formulario de autollenado.
3. Escriba el número de puntos en el círculo azul de la derecha y sume los puntos.
4. El resultado de la suma debe escribirlo en la parte inferior de este documento donde dice "total de puntos".

1. En el último año, ¿con qué frecuencia ha tomado alguna bebida alcohólica?

Registre el puntaje correspondiente según la respuesta del participante

Nunca	Una o menos veces al mes	2, 3 o 4 veces al mes	2 o 3 veces a la semana	4 o más veces a la semana
0 puntos	1 punto	2 puntos	3 puntos	4 puntos

2. ¿Cuántas copas, vasos, cervezas o tragos de bebidas alcohólicas suele tomar en un día de consumo normal?

Registre el puntaje correspondiente según la respuesta del participante

1 o 2	3 o 4	5 o 6	7, 8 o 9	10 o más
0 puntos	1 punto	2 puntos	3 puntos	4 puntos

3. ¿Con qué frecuencia toma seis (6) o más copas, vasos, cervezas o tragos de bebidas alcohólicas en un solo día?

Registre el puntaje correspondiente según la respuesta del participante

Nunca	Menos de una vez al mes	Mensualmente	Semanalmente	A diario o casi a diario
0 puntos	1 punto	2 puntos	3 puntos	4 puntos

4. En el último año, ¿con qué frecuencia fue difícil parar de tomar bebidas alcohólicas después de haber empezado?

Registre el puntaje correspondiente según la respuesta del participante

Nunca	Menos de una vez al mes	Mensualmente	Semanalmente	A diario o casi a diario
0 puntos	1 punto	2 puntos	3 puntos	4 puntos

5. En el último año, ¿con qué frecuencia no pudo hacer lo que se esperaba de usted porque había tomado bebidas alcohólicas?

Registre el puntaje correspondiente según la respuesta del participante

Nunca	Menos de una vez al mes	Mensualmente	Semanalmente	A diario o casi a diario
0 puntos	1 punto	2 puntos	3 puntos	4 puntos

6. En el último año, ¿con qué frecuencia ha necesitado tomar alguna bebida alcohólica en ayunas para recuperarse después de haber tomado mucho el día anterior?

Registre el puntaje correspondiente según la respuesta del participante

Nunca	Menos de una vez al mes	Mensualmente	Semanalmente	A diario o casi a diario
0 puntos	1 punto	2 puntos	3 puntos	4 puntos

7. En el último año, ¿con qué frecuencia ha tenido remordimientos o se ha sentido culpable después de haber tomado bebidas alcohólicas?

Registre el puntaje correspondiente según la respuesta del participante

Nunca	Menos de una vez al mes	Mensualmente	Semanalmente	A diario o casi a diario
0 puntos	1 punto	2 puntos	3 puntos	4 puntos

8. En el último año, ¿con qué frecuencia no ha podido recordar lo que sucedió la noche anterior porque había estado tomando bebidas alcohólicas?

Registre el puntaje correspondiente según la respuesta del participante

Nunca	Menos de una vez al mes	Mensualmente	Semanalmente	A diario o casi a diario
0 puntos	1 punto	2 puntos	3 puntos	4 puntos

9. ¿Usted o alguna otra persona ha resultado herida porque usted había tomado alguna bebida alcohólica?

Registre el puntaje correspondiente según la respuesta del participante

No	Sí, pero no en el curso del último año	Sí, en el último año
0 puntos	2 puntos	4 puntos

10. ¿Algún familiar, amigo, médico o personal de la salud ha mostrado preocupación por su forma de tomar bebidas alcohólicas o le han sugerido que deje de tomar?

Registre el puntaje correspondiente según la respuesta del participante

No	Sí, pero no en el curso del último año	Sí, en el último año
0 puntos	2 puntos	4 puntos

Total de puntos

Adaptación realizada por: Andrea López González, Danuby Adriana Buitrago López, Nelcy Rodríguez Malagón, Sebastián Patiño Rueda, Nicolás Martínes Ramos y Deyanira Sindy Moya Chaves

Fuente adaptada de: Babor TF, Higgins-Biddle JC, Saunders JB, Monteiro MG. AUDIT formulario de identificación de los transtornos debidos al consumo de alcohol [Internet]. s debidos al consumo de alcohol [Internet]. Organización Mundial de la Salud. Ginebra, Suiza; 2001 [citado el 14 de febrero de 2023]. Disponible en: https://www.paho.org/hq/dmdocuments/2009/AUDIT_spa.pdf. q/dmdocuments/2009/AUDIT_spa.pdf.

Published with permission from Pontificia Universidad Javeriana under a CC BY 4.0 license. Original copyright © 2024.

Versión entrevista

PARTE I

Instrucciones para persona encargada
A continuación, va a encontrar un formulario para identificar tempranamente a las personas que toman bebidas alcohólicas. Este formulario tiene dos versiones: una versión de autollenado que solo debe ser llenada por el participante y una versión entrevista que debe ser llenada junto con un personal de salud o agente social o investigador.

El formato que actualmente está leyendo es la versión entrevista. Siga los siguientes momentos:

MOMENTO 1

Tiene el formato de autollenado vacío

1. Lea lo siguiente al participante:
"las siguientes preguntas son sobre cómo usted toma bebidas alcohólicas. Necesitamos saber si esto está afectando su salud".

2. Haga la siguiente pregunta al participante:
"¿Aprendió a leer y escribir?".

- Sí sabe leer y escribir
  3. **Haga la siguiente pregunta**
  "¿Prefiere llenar el formulario solo o que una persona le ayude?".
  - Si prefiere llenar el formulario solo
    4. Entregue el formulario versión autollenado al participante
    Permita que responda las preguntas en privado
  - Si prefiere que una persona le ayude
    3. Inicie la entrevista (Siga la parte II)
- No sabe leer ni escribir el participante
  3. Inicie la entrevista (Siga la parte II)

MOMENTO 2

Tiene el formato de autollenado completo

1. Verifique lo siguiente
   - ✔ El formulario está lleno en su totalidad
     - ✖ si hace falta alguna respuesta, pregúntele al participante y complemente lo que falta
   - ✔ Cada pregunta solo tenga una respuesta
     - ✖ Si alguna pregunta tiene más de una respuesta, indíquele al participante que escoja solo una opción, la que mejor responda a la pregunta
2. Encuentre los puntos y escriba el valor en el círculo azul a la derecha de cada pregunta,
3. Sume los puntos de todas las preguntas
4. Escriba el resultado de la suma en el último cuadro en blanco donde dice "total de puntos"

Adaptación realizada por: Andrea López González, Danuby Adriana Buitrago López, Nelcy Rodríguez Malagón, Sebastián Patiño Rueda, Nicolás Martínes Ramos y Deyanira Sindy Moya Chaves

Fuente adaptada de: Babor TF, Higgins-Biddle JC, Saunders JB, Monteiro MG. AUDIT formulario de identificación de los transtornos debidos al consumo de alcohol [Internet]. Organización Mundial de la Salud. Ginebra, Suiza; 2001 [citado el 14 de febrero de 2023]. Disponible en: https://www.paho.org/hq/dmdocuments/2009/AUDIT_spa.pdf.

Published with permission from Pontificia Universidad Javeriana under a CC BY 4.0 license. Original copyright © 2024.

Código: ___________ Fecha: ___________

Versión entrevista

PARTE II

Los círculos de color azul deben ser llenados por la persona encargada

Los círculos de color blanco deben ser llenados con las respuestas del participante

Lea al participante:

"A continuación, le haré un formulario con 10 preguntas sobre cómo usted toma bebidas alcohólicas para saber si esto está afectando su salud. Le tomarán 10 minutos aproximadamente. Vamos a seguir 8 pasos y le mostraré 2 cuadros con imágenes para ayudarle a responder las preguntas. No hay respuestas buenas o malas. Responda con sinceridad. Una bebida alcohólica hace referencia a cualquier bebida comercial, casera o fermentada que contiene alcohol. Piense las veces que ha tomado alguna de estas bebidas, incluso para bajar la sed o para refrescarse. Recuerde cuando toma estas bebidas durante la semana o los fines de semana. Cuando decimos "en el último año", estamos hablando del tiempo que ha pasado desde hace 12 meses hasta hoy".

Escriba una ⓧ en los círculos con las respuestas del participante. Siga los pasos:

PASO 1

Muestre las siguientes imágenes al participante .
Explíquele al participante qué significa **en el último año** "Cuando decimos en el último año, estamos hablando del tiempo que ha pasado desde hace 12 meses hasta hoy".

Pregunte: ¿Cuál de las siguientes bebidas alcohólicas comerciales, caseras o fermentadas ha tomado en el último año? Escriba una ⓧ en los círculos con las respuestas del participante. Puede marcar más de una opción.

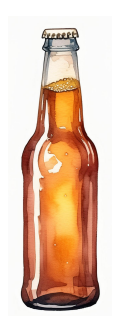
○ Cerveza, pola o coronita en botella

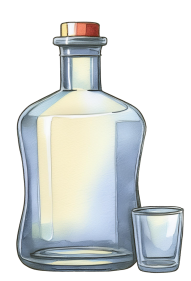
○ Aguardiente o guaro

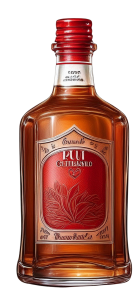
○ Ron

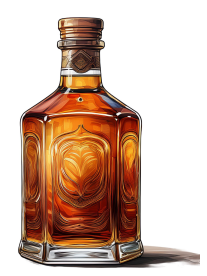
○ Whiskey

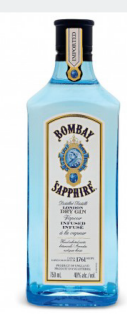
○ Ginebra

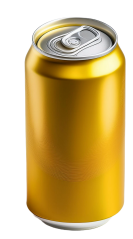
○ Cerveza, pola o coronita en lata

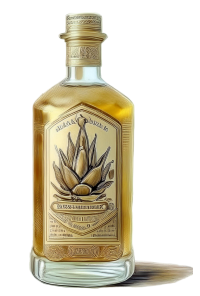
○ Tequila

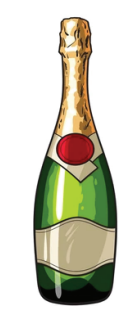
○ Champaña

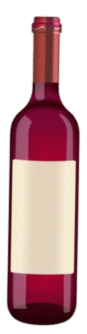
○ Vino

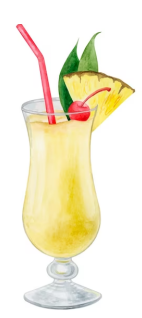
○ Cocteles piña colada

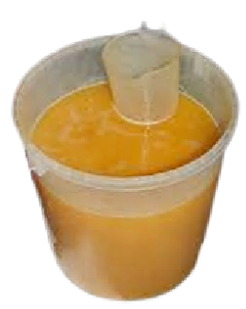
○ Guarapo

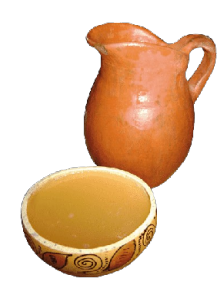
○ Chicha

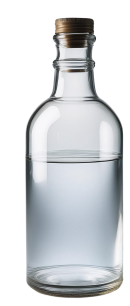
○ Viche

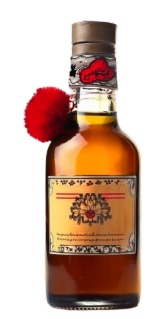
○ Chirrinchi

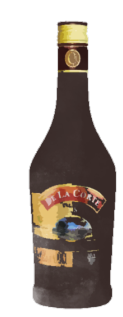
○ Crema de Whiskey

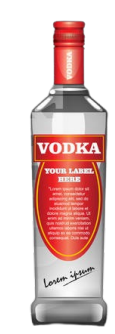
○ Vodka

PASO 2

Recuerde la definición de bebida alcohólica y lo que el participante marcó en el paso 1. **Lea lo siguiente al participante:** **"**Piense las veces que ha tomado alguna de estas bebidas, incluso para bajar la sed o para refrescarse. Recuerde cuando toma estas bebidas durante la semana o los fines de semana. Usted me acaba de contar que en el último año ha tomado...".

1. Lea la siguiente pregunta: en el último año, ¿con qué frecuencia ha tomado alguna bebida alcohólica? Escriba una ⓧ en el círculo con la respuesta del participante. Solo puede marcar una opción.

○	A. Nunca	0 puntos
○	B. Una o menos veces al mes	1 punto
○	C. 2 o 4 veces al mes	2 puntos
○	D. 2 o 3 veces a la semana	3 puntos
○	E. 4 o más veces a la semana	4 puntos

Puntaje
Registre el puntaje correspondiente según la respuesta

Adaptación realizada por: Andrea López González, Danuby Adriana Buitrago López, Nelcy Rodríguez Malagón, Sebastián Patiño Rueda, Nicolás Martínes Ramos y Deyanira Sindy Moya Chaves
Fuente adaptada de: Babor TF, Higgins-Biddle JC, Saunders JB, Monteiro MG. AUDIT formulario de identificación de los transtornos debidos al consumo de alcohol [Internet]. Organización Mundial de la Salud. Ginebra, Suiza; 2001 [citado el 14 de febrero de 2023]. Disponible en: https://www.paho.org/hq/dmdocuments/2009/AUDIT_spa.pdf.

Published with permission from Pontificia Universidad Javeriana under a CC BY 4.0 license. Original copyright © 2024.

Versión entrevista

PASO 3

Si la respuesta del participante fue la **A** en la pregunta (1), continúe a las preguntas (9) y (10). Las preguntas (2) a (8) no aplican.
Si la respuesta fue **B, C, D O E**, continúe con la pregunta (2)

PASO 4

Muestre las siguientes imágenes al participante.

Pregunte: ¿Cuál de los siguientes recipientes usa para tomar bebidas alcohólicas comerciales, caseras o fermentadas?
Escriba una (X) en el círculo con la respuesta del participante. Puede marcar más de una opción.

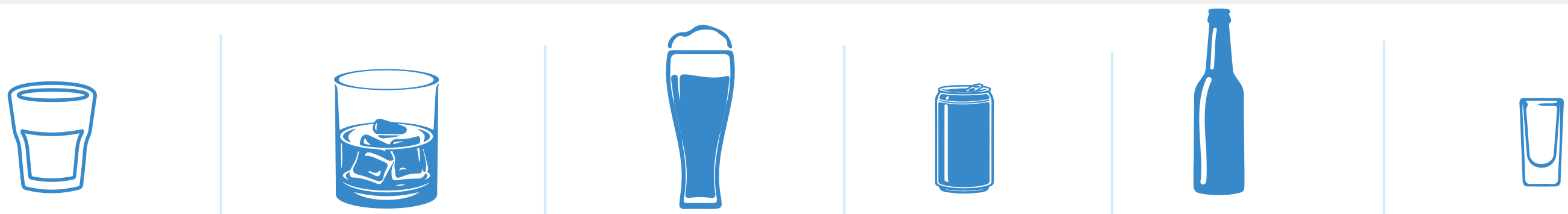

○ Vaso pequeño
○ Vaso mediano
○ Vaso grande
○ Lata
○ Botella
○ Copa delgada

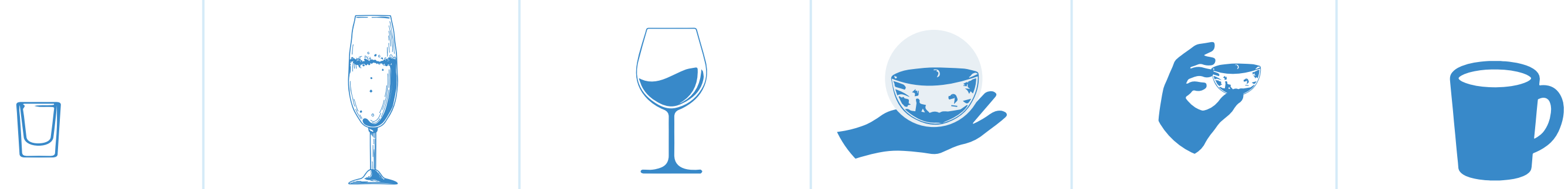

○ Copa pequeña
○ Copa larga
○ Copa Grande
○ Totuma grande
○ Totuma pequeña
○ Pocillo

PASO 5

Recuerde la definición de bebida alcohólica y lo que el participante marcó en el paso 1 y 4.
Recuerde la definición de consumo normal **"Piense las veces que ha tomado alguna de estas bebidas, incluso para bajar la sed o para refrescarse. Recuerde cuando toma estas bebidas durante la semana o los fines de semana".**

2. Lea la siguiente pregunta: ¿Cuántas copas, vasos, cervezas o tragos de bebidas alcohólicas suele tomar en un día de consumo normal?
Escriba una (X) en el círculo con la respuesta del participante. Solo puede marcar una opción.

○	A. 1 o 2 copas, vasos, cervezas o tragos	0 puntos
○	B. 3 o 4 copas, vasos, cervezas o tragos	1 punto
○	C. 5 o 6 copas, vasos, cervezas o tragos	2 puntos
○	D. 7, 8 o 9 copas, vasos, cervezas o tragos	3 puntos
○	E. 10 o más copas, vasos, cervezas o tragos	4 puntos

Puntaje
Registre el puntaje correspondiente según la respuesta

Adaptación realizada por: Andrea López González, Danuby Adriana Buitrago López, Nelcy Rodríguez Malagón, Sebastián Patiño Rueda, Nicolás Martínes Ramos y Deyanira Sindy Moya Chaves
Fuente adaptada de: Babor TF, Higgins-Biddle JC, Saunders JB, Monteiro MG. AUDIT formulario de identificación de los transtornos debidos al consumo de alcohol [Internet]. Organización Mundial de la Salud. Ginebra, Suiza; 2001 [citado el 14 de febrero de 2023]. Disponible en: https://www.paho.org/hq/dmdocuments/2009/AUDIT_spa.pdf.

Published with permission from Pontificia Universidad Javeriana under a CC BY 4.0 license. Original copyright © 2024.

Versión entrevista

PASO 6

Las siguientes preguntas tienen estas opciones de respuesta:
- Nunca que significa ni una sola vez en el último año.
- Menos de una vez al mes que significa cada 2 meses, cada 3 meses, cada 4 meses, cada 5 meses, cada 6 meses, cada 7 meses, cada 8 meses, cada 9 meses, cada 10 meses, cada 11 meses, cada 12 meses.
- Mensualmente que significa cada mes.
- Semanalmente que significa cada semana.
- A diario o casi a diario que significa todos los días o casi todos los días.

¡Recuerde la definición de bebida alcohólica!

3. Lea la siguiente pregunta: ¿con qué frecuencia toma **seis (6) o más** copas, vasos, cervezas o tragos de bebidas alcohólicas en un solo día? Escriba una ⓧ en el círculo con la respuesta del participante. Solo puede marcar una opción.

○	A. Nunca	0 puntos
○	B. Menos de una vez al mes	1 punto
○	C. Mensualmente	2 puntos
○	D. Semanalmente	3 puntos
○	E. A diario o casi a diario	4 puntos

Puntaje
Registre el puntaje correspondiente según la respuesta

PASO 7

Sume el puntaje de las preguntas 2 y 3
Si esta suma es igual a **0 puntos**, continúe a la pregunta 9 y 10. Las preguntas 4 a 8 no aplican.
Si esta suma es igual o mayor a **1 punto**, continúe con la pregunta 4
Si continúa con la pregunta 4, explíquele al participante qué significa en el último año. "Cuando decimos en el último año, estamos hablando del tiempo que ha pasado desde hace 12 meses hasta hoy".

4. Lea la siguiente pregunta: en el último año, ¿con qué frecuencia fue difícil parar de tomar bebidas alcohólicas después de haber empezado?
Escriba una ⓧ en el círculo con la respuesta del participante. Solo puede marcar una opción.

○	A. Nunca	0 puntos
○	B. Menos de una vez al mes	1 punto
○	C. Mensualmente	2 puntos
○	D. Semanalmente	3 puntos
○	E. A diario o casi a diario	4 puntos

Puntaje
Registre el puntaje correspondiente según la respuesta

5. Lea la siguiente pregunta: en el último año, ¿con qué frecuencia no pudo hacer lo que se esperaba de usted porque había tomado bebidas alcohólicas?
Escriba una ⓧ en el círculo con la respuesta del participante. Solo puede marcar una opción.

○	A. Nunca	0 puntos
○	B. Menos de una vez al mes	1 punto
○	C. Mensualmente	2 puntos
○	D. Semanalmente	3 puntos
○	E. A diario o casi a diario	4 puntos

Puntaje
Registre el puntaje correspondiente según la respuesta

Published with permission from Pontificia Universidad Javeriana under a CC BY 4.0 license. Original copyright © 2024.

Versión entrevista

6. Lea la siguiente pregunta: en el último año, ¿con qué frecuencia ha necesitado tomar alguna bebida alcohólica en ayunas para recuperarse después de haber tomado mucho el día anterior?
Escriba una ⓧ en el círculo con la respuesta del participante. Solo puede marcar una opción.

○	A. Nunca	0 puntos
○	B. Menos de una vez al mes	1 punto
○	C. Mensualmente	2 puntos
○	D. Semanalmente	3 puntos
○	E. A diario o casi a diario	4 puntos

Puntaje
Registre el puntaje correspondiente según la respuesta

7. Lea la siguiente pregunta: en el último año, ¿con qué frecuencia ha tenido remordimientos o se ha sentido culpable después de haber tomado bebidas alcohólicas?
Escriba una ⓧ en el círculo con la respuesta del participante. Solo puede marcar una opción.

○	A. Nunca	0 puntos
○	B. Menos de una vez al mes	1 punto
○	C. Mensualmente	2 puntos
○	D. Semanalmente	3 puntos
○	E. A diario o casi a diario	4 puntos

Puntaje
Registre el puntaje correspondiente según la respuesta

8. Lea la siguiente pregunta: en el último año, ¿con qué frecuencia no ha podido recordar lo que sucedió la noche anterior porque había estado tomando bebidas alcohólicas?
Escriba una ⓧ en el círculo con la respuesta del participante. Solo puede marcar una opción.

○	A. Nunca	0 puntos
○	B. Menos de una vez al mes	1 punto
○	C. Mensualmente	2 puntos
○	D. Semanalmente	3 puntos
○	E. A diario o casi a diario	4 puntos

Puntaje
Registre el puntaje correspondiente según la respuesta

PASO 8

Explíquele al participante qué significa **en el último año**. “Cuando decimos **en el último año**, estamos hablando del tiempo que ha pasado desde hace 12 meses hasta hoy”.

9. Lea la siguiente pregunta: ¿usted o alguna otra persona ha resultado herido porque usted había tomado alguna bebida alcohólica?
Escriba una ⓧ en el círculo con la respuesta del participante. Solo puede marcar una opción.

○	A. No	0 puntos
○	B. Sí, pero no en el curso del último año	2 puntos
○	C. Sí, en el último año	4 puntos

Puntaje
Registre el puntaje correspondiente según la respuesta

Published with permission from Pontificia Universidad Javeriana under a CC BY 4.0 license. Original copyright © 2024.

Código: ___________ Fecha: ___________

Versión entrevista

10. Lea la siguiente pregunta: ¿Algún familiar, amigo, médico o personal de la salud ha mostrado preocupación por su forma de tomar bebidas alcohólicas o le han sugerido que deje de tomar?
Escriba una (X) en el círculo con la respuesta del participante. Solo puede marcar una opción.

○	A. No	0 puntos
○	B. Sí, pero no en el curso del último año	2 puntos
○	C. Sí, en el último año	4 puntos

Puntaje
Registre el puntaje correspondiente según la respuesta

Sume los puntos obtenidos por el participante en cada pregunta y regístrelo acá:

Adaptación realizada por: Andrea López González, Danuby Adriana Buitrago López, Nelcy Rodríguez Malagón, Sebastián Patiño Rueda, Nicolás Martínes Ramos y Deyanira Sindy Moya Chaves
Fuente adaptada de: Babor TF, Higgins-Biddle JC, Saunders JB, Monteiro MG. AUDIT formulario de identificación de los transtornos debidos al consumo de alcohol [Internet]. Organización Mundial de la Salud. Ginebra, Suiza; 2001 [citado el 14 de febrero de 2023]. Disponible en: https://www.paho.org/hq/dmdocuments/2009/AUDIT_spa.pdf.

Published with permission from Pontificia Universidad Javeriana under a CC BY 4.0 license. Original copyright © 2024.

Ficha No. 1

Una bebida alcohólica hace referencia a cualquier bebida comercial, casera o fermentada que contiene alcohol.

¡Recuerde! Existen muchos tipos de bebidas alcohólicas como:

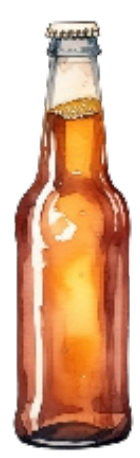
Cerveza, pola o coronita en botella

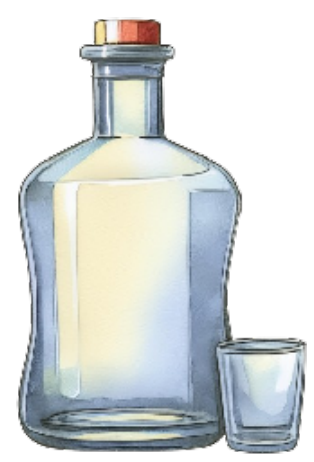
Aguardiente o guaro

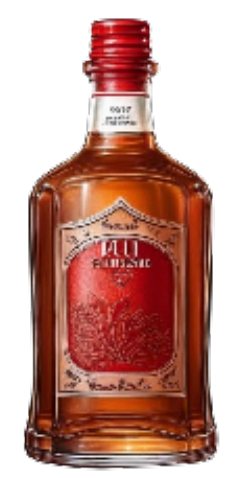
Ron

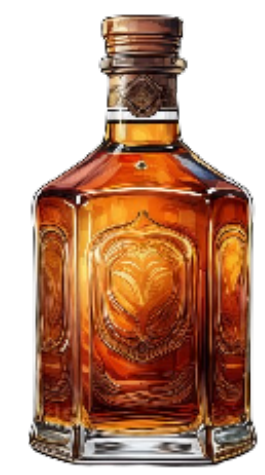
Whiskey

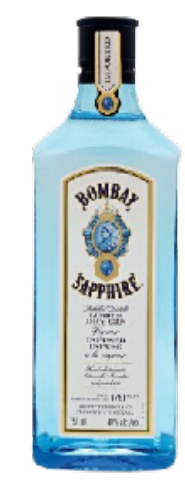

Ginebra

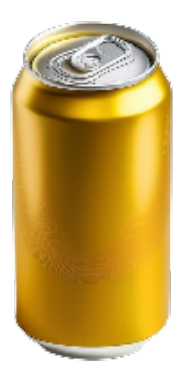
Cerveza, pola o coronita en botella

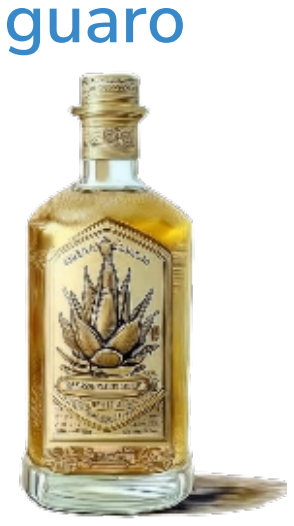
Tequila

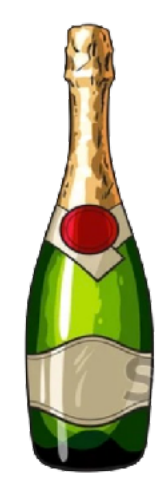
Champaña

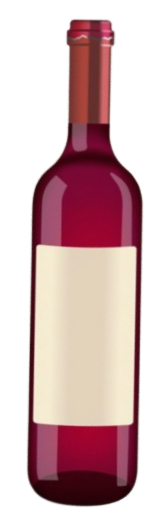
Vino

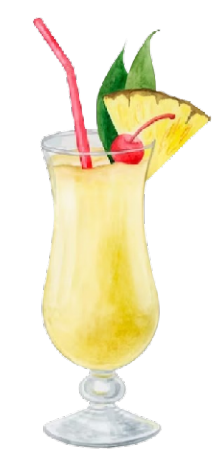
Cocteles piña colada

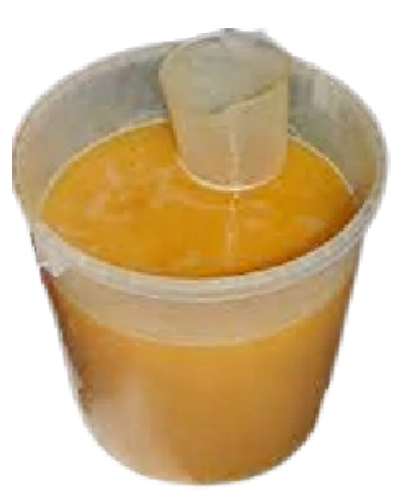
Guarapo

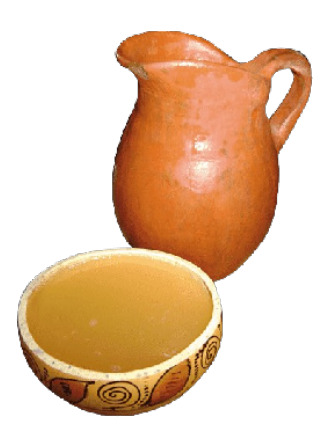
Chicha

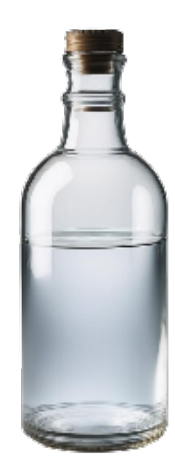
Viche

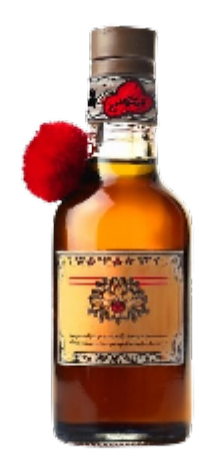
Chirrinchi

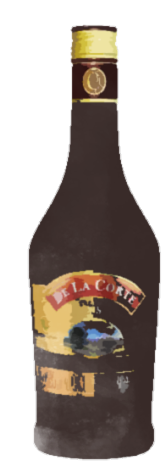
Crema de Whiskey

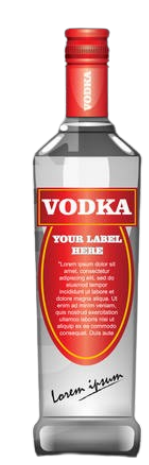

Vodka

Entregue esta ficha al participante para ayudarle a responder las preguntas 1 y 2

Adaptación realizada por: Andrea López González, Danuby Adriana Buitrago López, Nelcy Rodríguez Malagón, Sebastián Patiño Rueda, Nicolás Martínes Ramos y Deyanira Sindy Moya Chaves

Fuente adaptada de: Babor TF, Higgins-Biddle JC, Saunders JB, Monteiro MG. AUDIT formulario de identificación de los transtornos debidos al consumo de alcohol [Internet]. Organización Mundial de la Salud. Ginebra, Suiza; 2001 [citado el 14 de febrero de 2023]. Disponible en: https://www.paho.org/hq/dmdocuments/2009/AUDIT_spa.pdf.

Published with permission from Pontificia Universidad Javeriana under a CC BY 4.0 license. Original copyright © 2024.

Ficha No. 2

Una bebida alcohólica hace referencia a cualquier bebida comercial, casera o fermentada que contiene alcohol.

¡Recuerde! Existen muchos recipientes para tomar bebidas alcohólicas, por ejemplo:

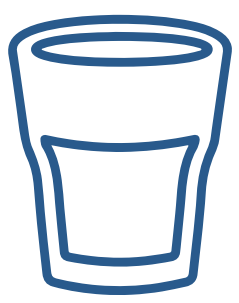
Vaso pequeño

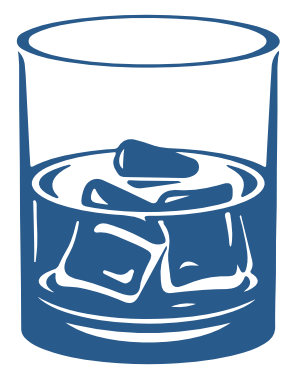
Vaso mediano

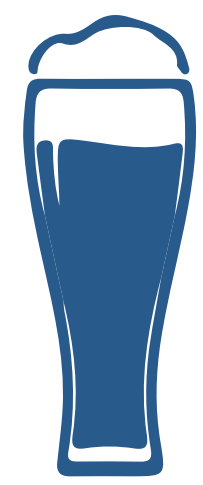
Vaso grande

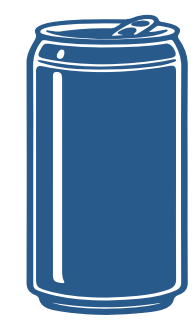
Lata

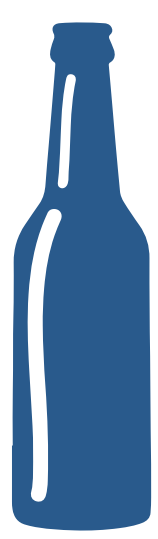
Botella

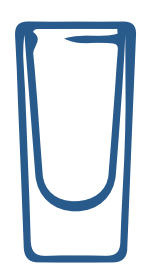
Copa delgada

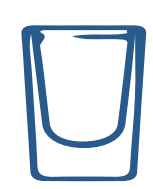
Copa pequeña

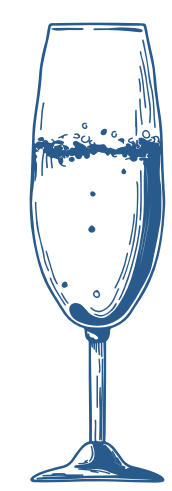
Copa larga

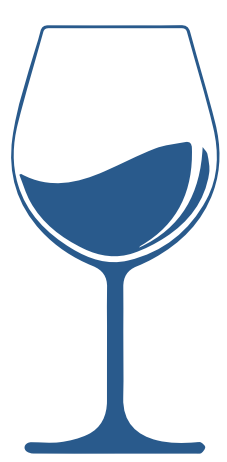
Copa grande

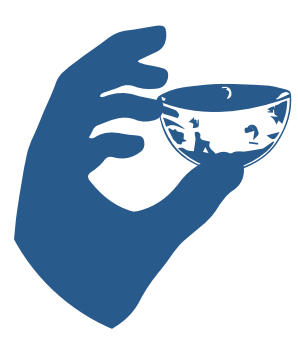
Totuma pequeña

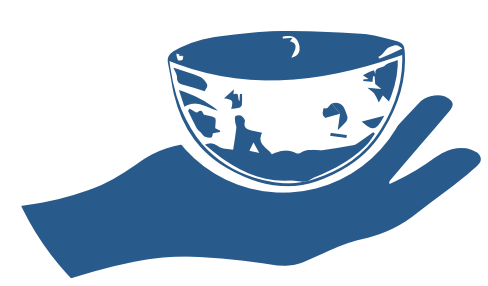
Totuma grande

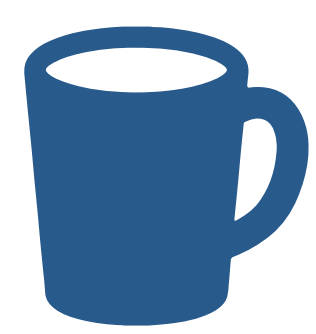
Pocillo

Entregue esta ficha al participante para ayudarle a responder la pregunta 3

Adaptación realizada por: Andrea López González, Danuby Adriana Buitrago López, Nelcy Rodríguez Malagón, Sebastián Patiño Rueda, Nicolás Martínes Ramos y Deyanira Sindy Moya Chaves

Fuente adaptada de: Babor TF, Higgins-Biddle JC, Saunders JB, Monteiro MG. AUDIT formulario de identificación de los transtornos debidos al consumo de alcohol [Internet]. Organización Mundial de la Salud. Ginebra, Suiza; 2001 [citado el 14 de febrero de 2023]. Disponible en: https://www.paho.org/hq/dmdocuments/2009/AUDIT_spa.pdf.

Published with permission from Pontificia Universidad Javeriana under a CC BY 4.0 license. Original copyright © 2024.
