# Supplementary material for: Strengthening alcohol consumption screening in primary care for culturally diverse populations in Latin America: Adaptation and content and face validity of the AUDIT in Guaviare, Colombia
Source: PLoS One. 2026 Sep 30;21(9):e0359625. doi: 10.1371/journal.pone.0359625 (PMC13626350; doi:10.1371/journal.pone.0359625)
Supplement: Supplementary file 2 [file pone.0359625.s001.docx]

**Original AUDIT Versions**

This appendix includes the Spanish version of the Alcohol Use Disorders Identification Test (AUDIT), published by the World Health Organization (WHO) in 2001 for use in Latin America, which served as the source instrument for the cultural adaptation conducted in Guaviare, Colombia. Both the interviewer-administered and self-administered formats are included. The original English version is also provided for reference to facilitate interpretation by international readers.

**AUDIT self-administered format: Original Spanish version.**

| **Test de Identificación de Trastornos por consumo de Alcohol: versión de auto-pase.**  PACIENTE: Debido a que el uso del alcohol puede afectar su salud e interferir con ciertos medicamentos y tratamientos, es importante que le hagamos algunas preguntas sobre su uso del alcohol. Sus respuestas serán confidenciales, así que sea honesto por favor.  Marque una X en el cuadro que mejor describa su respuesta a cada pregunta.  **The Alcohol Use Disorders Identification Test: Self-Report Version**  PATIENT: Because alcohol use can affect your health and can interfere with certain medications and treatments, it is important that we ask some questions about your use of alcohol. Your answers will remain confidential so please be honest.  Place an X in one box that best describes your answer to each question. | | | | | |
| --- | --- | --- | --- | --- | --- |
| Preguntas | 0 | 1 | 2 | 3 | 4 |
| 1. ¿Con qué frecuencia consume alguna bebida alcohólica? | Nunca | Una o menos veces al mes | De 2 a 4 veces al mes | De 2 a 3 más veces a la semana | 4 o más veces a la semana |
| 1. How often do you have a drink containing alcohol? | Never | Monthly or les | 2-4 times a month | 2-3 times a week | 4 or more times a week |
| 2. ¿Cuántas consumiciones de bebidas alcohólicas suele realizar en un día de consumo normal? | 1 o 2 | 3 o 4 | 5 o 6 | De 7 a 9 | 10 o más |
| 2. How many drinks containing alcohol do you have on a typical  day when you are drinking? | 1 or 2 | 3 or 4 | 5 or 6 | 7 to 9 | 10 or more |
| 3. ¿Con qué frecuencia toma 6 o más bebidas alcohólicas en un solo día? | Nunca | Menos de una vez al mes | Mensualmente | Semanalmente | A diario o casi a diario |
| 3. How often do you have six or more drinks on one occasion? | Never | Less tan monthly | Monthly | Weekly | Daily or almost daily |
| 4. ¿Con qué frecuencia en el curso del último año ha sido incapaz de parar de beber una vez había empezado? | Nunca | Menos de una vez al mes | Mensualmente | Semanalmente | A diario o casi a diario |
| 4. How often during the last year have you found that you were not able to stop drinking once you had started? | Never | Less tan monthly | Monthly | Weekly | Daily or almost daily |
| 5. Con qué frecuencia en el curso del último año no pudo hacer lo que se esperaba de usted porque había bebido? | Nunca | Menos de una vez al mes | Mensualmente | Semanalmente | A diario o casi a diario |
| 5. How often during the last year have you failed to do what was normally expected of you because of drinking? | Never | Less tan monthly | Monthly | Weekly | Daily or almost daily |
| 6. ¿Con qué frecuencia en el curso del último año ha necesitado beber en ayunas para recuperarse después de haber bebido mucho el día anterior? | Nunca | Menos de una vez al mes | Mensualmente | Semanalmente | A diario o casi a diario |
| 6. How often during the last year have you needed a first drink in the morning to get yourself going after a heavy drinking session? | Never | Less tan monthly | Monthly | Weekly | Daily or almost daily |
| 7. ¿Con qué frecuencia en el curso del último año ha tenido remordimientos o sentimientos de culpa después de haber bebido? | Nunca | Menos de una vez al mes | Mensualmente | Semanalmente | A diario o casi a diario |
| 7. How often during the last year have you had a feeling of guilt or remorse after drinking? | Never | Less tan monthly | Monthly | Weekly | Daily or almost daily |
| 8. ¿Con qué frecuencia en el curso del último año no ha podido recordar lo que sucedió la noche anterior porque había estado bebiendo? | Nunca | Menos de una vez al mes | Mensualmente | Semanalmente | A diario o casi a diario |
| 8. How often during the last year have you been unable to remember what happened the night before because of your drinking? | Never | Less tan monthly | Monthly | Weekly | Daily or almost daily |
| 9. ¿Usted o alguna otra persona ha resultado herido porque usted había bebido? | No |  | Sí, pero no en el curso del último año |  | Sí, el último año |
| 9. Have you or someone else been injured because of your drinking? | No |  | Yes, but not in the last year |  | Yes, during the last year |
| 10. ¿Algún familiar, amigo, médico o profesional sanitario ha mostrado preocupación por un consumo de bebidas alcohólicas o le han sugerido que deje de beber? | No |  | Sí, pero no en el curso del último año |  | Sí, el último año |
| 10. Has a relative, friend, doctor, or other health care worker been concerned about your drinking or suggested you cut down? | No |  | Yes, but not in the last year |  | Yes, during the last year |
|  |  |  |  |  | Total |

**Source:** *Spanish version:* Babor TF, Higgins-Biddle JC, Saunders JB, Monteiro MG. AUDIT: Cuestionario de identificación de los transtornos debidos al consumo de alcohol [Internet]. Organización Mundial de la Salud. Geneva, Switzerland; 2001 [accessed 14 February 2023]. Available at: <https://www.paho.org/sites/default/files/AUDIT_spa.pdf>. *Original English version:* Babor TF, Higgins-Biddle JC, Saunders JB, Monteiro MG. AUDIT: The Alcohol Use Disorders Identification Test: Guidelines for Use in Primary Care [Internet]. World Health Organization. Geneva, Switzerland; 2001 [accessed 27 July 2026]. Available at: <https://iris.who.int/server/api/core/bitstreams/c57d9855-5450-4c46-84b1-c88a6df4192c/content>

**AUDIT interview format: Original Spanish version.**

| **Test de Identificación de Trastornos por consumo de Alcohol: versión entrevista.**  Lea las preguntas tal y como están escritas. Registre las respuestas cuidadosamente. Empiece el AUDIT diciendo “Ahora voy a hacerle algunas preguntas sobre su consumo de bebidas alcohólicas durante el último año”. Explique qué entiende por “bebidas alcohólicas” utilizando ejemplos típicos como cerveza, vino, vodka, etc. Codifique las respuestas en términos de consumiciones (“bebidas estándar”). Marque la cifra de la respuesta adecuada en el recuadro de la derecha.  **The Alcohol Use Disorders Identification Test: Interview Version**  Read questions as written. Record answers carefully. Begin the AUDIT by saying “Now I am going to ask you some questions about your use of alcoholic beverages during this past year.” Explain what is meant by “alcoholic beverages” by using local examples of beer, wine, vodka, etc. Code answers in terms of “standard drinks”. Place the correct answer number in the box at the right. | |
| --- | --- |
| 1. ¿Con qué frecuencia consume alguna bebida alcohólica?   (0) Nunca (Pase a las preguntas 9 – 10) (1) Una o menos veces al mes (2) De 2 a 4 veces al mes (3) De 2 a 3 veces a la semana (4) 4 o más veces a la semana | 6. ¿Con qué frecuencia en el curso del último año ha necesitado beber en ayunas para recuperarse después de haber bebido mucho el día anterior?   (0) Nunca (1) Menos de una vez al mes (2) Mensualmente (3) Semanalmente (4) A diario o casi a diario |
| 1. How often do you have a drink containing alcohol?  (0) Never [Skip to Qs 9-10]  (1) Monthly or less  (2) 2 to 4 times a month  (3) 2 to 3 times a week  (4) 4 or more times a week | 6. How often during the last year have you needed  a first drink in the morning to get yourself going  after a heavy drinking session?  (0) Never  (1) Less than monthly  (2) Monthly  (3) Weekly  (4) Daily or almost daily |
| 2. ¿Cuántas consumiciones de bebidas alcohólicas suele realizar en un día de consumo normal?  (0) 1 o 2  (1) 3 o 4  (2) 5 o 6  (3) 7, 8 o 9  (4) 10 o más | 7. ¿Con qué frecuencia en el curso del último año ha tenido remordimientos o sentimientos de culpa después de haber bebido?   (0) Nunca (1) Menos de una vez al mes (2) Mensualmente (3) Semanalmente (4) A diario o casi a diario |
| 2. How many drinks containing alcohol do you have  on a typical day when you are drinking?  (0) 1 or 2  (1) 3 or 4  (2) 5 or 6  (3) 7, 8, or 9  (4) 10 or more | 7. How often during the last year have you had a  feeling of guilt or remorse after drinking?  (0) Never  (1) Less than monthly  (2) Monthly  (3) Weekly  (4) Daily or almost daily |
| 3. ¿Con qué frecuencia toma 6 o más bebidas alcohólicas en un solo día?   (0) Nunca (1) Menos de una vez al mes (2) Mensualmente (3) Semanalmente (4) A diario o casi a diario   Pase a las preguntas 9 y 10 si la suma total de las preguntas 2 y 3 es = 0 | 8. ¿Con qué frecuencia en el curso del último año no ha podido recordar lo que sucedió la noche anterior porque había estado bebiendo?   (0) Nunca (1) Menos de una vez al mes (2) Mensualmente (3) Semanalmente (4) A diario o casi a diario |
| 3. How often do you have six or more drinks on one  occasion?  (0) Never  (1) Less than monthly  (2) Monthly  (3) Weekly  (4) Daily or almost daily  Skip to Questions 9 and 10 if Total Score  for Questions 2 and 3 = 0 | 8. How often during the last year have you been  unable to remember what happened the night  before because you had been drinking?  (0) Never  (1) Less than monthly  (2) Monthly  (3) Weekly  (4) Daily or almost daily |
| 4. ¿Con qué frecuencia en el curso del último año ha sido incapaz de parar de beber una vez había empezado?   (0) Nunca (1) Menos de una vez al mes (2) Mensualmente (3) Semanalmente (4) A diario o casi a diario | 9. ¿Usted o alguna otra persona ha resultado herido porque usted había bebido?  (0) No  (2) Sí, pero no en el curso del último año  (4) Sí, en el último año |
| 4. How often during the last year have you found  that you were not able to stop drinking once you  had started?  (0) Never  (1) Less than monthly  (2) Monthly  (3) Weekly  (4) Daily or almost daily | 9. Have you or someone else been injured as a  result of your drinking?  (0) No  (2) Yes, but not in the last year  (4) Yes, during the last year |
| 5. ¿Con qué frecuencia en el curso del último año no pudo hacer lo que se esperaba de usted porque había bebido?   (0) Nunca (1) Menos de una vez al mes (2) Mensualmente (3) Semanalmente (4) A diario o casi a diario | 10. ¿Algún familiar, amigo, médico o profesional sanitario ha mostrado preocupación por un consumo de bebidas alcohólicas o le han sugerido que deje de beber?  (0) No  (2) Sí, pero no en el curso del último año  (4) Sí, en el último año |
| 5. How often during the last year have you failed to do what was normally expected from you because of drinking?  (0) Never  (1) Less than monthly  (2) Monthly  (3) Weekly  (4) Daily or almost daily | 10. Has a relative or friend or a doctor or another  health worker been concerned about your drinking or suggested you cut down?  (0) No  (2) Yes, but not in the last year  (4) Yes, during the last year |
| **Registre la puntuación total aquí**  **Record total of specific items here** | |

**Source:** *Spanish version:* Babor TF, Higgins-Biddle JC, Saunders JB, Monteiro MG. AUDIT: Cuestionario de identificación de los transtornos debidos al consumo de alcohol [Internet]. Organización Mundial de la Salud. Geneva, Switzerland; 2001 [accessed 14 February 2023]. Available at: <https://www.paho.org/sites/default/files/AUDIT_spa.pdf>. *Original English version:* Babor TF, Higgins-Biddle JC, Saunders JB, Monteiro MG. AUDIT: The Alcohol Use Disorders Identification Test: Guidelines for Use in Primary Care [Internet]. World Health Organization. Geneva, Switzerland; 2001 [accessed 27 July 2026]. Available at: <https://iris.who.int/server/api/core/bitstreams/c57d9855-5450-4c46-84b1-c88a6df4192c/content>
