# Supplementary material for: Strengthening alcohol consumption screening in primary care for culturally diverse populations in Latin America: Adaptation and content and face validity of the AUDIT in Guaviare, Colombia
Source: PLoS One. 2026 Sep 30;21(9):e0359625. doi: 10.1371/journal.pone.0359625 (PMC13626350; doi:10.1371/journal.pone.0359625)
Supplement: Supplementary file 3 [file pone.0359625.s002.docx]

**Structured Observation Guides**

| **Structured Observation Guide**    Observation number: ______    **Project:** Cultural adaptation and psychometric validation of the AUDIT scale for screening hazardous alcohol consumption in an adult population in Guaviare | |
| --- | --- |
| **Why are we doing this?**  We seek to describe and understand alcohol consumption patterns in the communities of Guaviare. This will help us better understand how to adjust the tool we use to identify individuals with hazardous alcohol consumption. We hope that after making these adjustments, the tool can be used in primary care settings. | |
| **How to prepare for the observation?**   - Have a black pen. - Print the guide. - Allow approximately 20–30 minutes of your time. - Travel to the location where you will conduct the observation. - Choose any day of the week or weekend to conduct the observation.   You must complete 5 observations. Each observation must be conducted in a different location and on a different day.   - Remember, this is an observation process, you should not ask questions to the people at the location. What you must do is answer the following questions based on what you observe. All questions must be answered. | |
| **Step 2:**  Who conducts the observation?  [Write your name in the blank space] | _________________________________ |
| **Step 3:**  In which location in Guaviare did you conduct the observation?  [Mark with an X the letter that corresponds to your answer, one answer only] | a. San José del Guaviare b. Boquerón |
| **Step 4:**  Where were you when conducting the observation?  [Mark with an X the letter that corresponds to your answer, one answer only] | a. Store b. Cantina or bar c. Community member's home d. Tejo courts^1^ e. Pool hall f. Farm |
| **Step 5:**  Are people drinking alcohol in this location?  [Mark with an X the letter that corresponds to your answer, one answer only] | a. Yes b. No |
| **Step 6:**  Are traditional beverages produced in this location (for example: guarapo, chicha, pipirí)?  [Mark with an X the letter that corresponds to your answer, one answer only] | a. Yes b. No |
| **Step 7:**  If traditional beverages are produced in this location, which one is produced?  [Write the name of the traditional beverage that is produced] | _____________________ |
| **Step 8:**  In what context are people drinking alcohol?  [Mark with an X the letter that corresponds to your answer, you may mark more than one answer] | a. Community gatherings or celebrations b. Playing tejo^1^ c. During work hours d. Family gathering e. Playing pool f. Playing soccer g. Other. Which? _______________ |
| **Step 9:**  When did you conduct the observation?  [Write the date in numbers. Example: 03/21/2024] | _____ / _____ / ________  Day    /   Month /    Year |
| **Step 10:**  What time did the observation start?  [Write the time in standard format. Example: 1 in the afternoon would be 1 p.m. 6 in the morning would be 6 a.m.] | _______ |
| **Step 11:**  What time did the observation end?  [Write the time in standard format. Example: 1 in the afternoon would be 1 p.m. 6 in the morning would be 6 a.m.] | _______ |
| **Step 12:**  How many people were present at the location?  [Write the number of people] | _____  people |
| **Step 13:**  Of the people present at the location, how many were drinking alcohol?  [Write the number of people] | _____  people |
| **Step 14:**  Based on what you heard, how do community members refer to alcohol?  *(Note: options a–d are different colloquial terms for alcoholic drink in Spanish)* | a. Trago b. Licor c. Bebida de alcohol d. Other.Which? ______________ |
| **Step 15:**  What type of alcohol were people drinking?  [Mark with an X the letter that corresponds to your answer, you may mark more than one answer] | a. Beer b. Rum c. Aguardiante^2^ d. Whiskey e. Vodka f. Tequila g. Guarapo^3^ h. Chicha^3^ i. Viche^3^ j. Pipirí (fermented chontaduro beverage)^3^ k. Other. Which? ________________ |
| **Step 16:**  Of the alcoholic beverages being consumed, which one did you observe most frequently?  [Mark with an X the letter that corresponds to your answer, one answer only] | a. Beer b. Rum c. Aguardiante^2^ d. Whiskey e. Vodka f. Tequila g. Guarapo^3^ h. Chicha^3^ i. Viche^3^ j. Pipirí (fermented chontaduro beverage)^3^ k. Other. Which? ________________ |
| **Step 17:**  Of the people you recognize who were drinking alcohol, how frequently do they usually do so?  [Mark with an X the letter that corresponds to your answer, one answer only] | a. Never  b. Monthly or less  c. 2 to 4 times a month  d. 2 to 3 times a week  e. 4 or more times a week |
| **Step 18:**  Based on your observation, approximately how many drinks was each person consuming?  [Mark with an X the letter that corresponds to your answer, one answer only] | a. 1 or 2 b. 3 or 4 c. 5 or 6 d. 7 or 9 e. 10 or more |
| **Step 19:**  Based on your observation, what container were people using to drink alcohol?  [Mark with an X the letter that corresponds to your answer, you may mark more than one answer] | a. Bottle  b. Glass  c. Wine glass  d. Totuma^4^  e. Large cup  f. Other. Which? ___________________ |

Source: own elaboration

^1^ Tejo is a traditional Colombian sport in which players throw a metal disc toward a clay-filled target, typically played in social settings.

^2^ Aguardiente is a traditional Latin American anise-flavored distilled liquor.

^3^ Traditional fermented or distilled beverages commonly consumed in the Guaviare region of Colombia.

^4^ Traditional drinking vessel made from the dried shell of the totumo fruit (Crescentia cujete), widely used in rural and indigenous communities in Colombia
