# Supplementary material for: Strengthening alcohol consumption screening in primary care for culturally diverse populations in Latin America: Adaptation and content and face validity of the AUDIT in Guaviare, Colombia
Source: PLoS One. 2026 Sep 30;21(9):e0359625. doi: 10.1371/journal.pone.0359625 (PMC13626350; doi:10.1371/journal.pone.0359625)
Supplement: Supplementary file 4 [file pone.0359625.s003.docx]

**Nominal Group Technique Guide**

| **Project:** Cultural adaptation and psychometric validation of the AUDIT scale for screening hazardous alcohol consumption in an adult population in Guaviare.  **Nominal Group Technique Guide**  **Community Expert Group** | | |
| --- | --- | --- |
| **Team** | | |
|  | *Names* | *Roles* |
| **Facilitator 1** | Andrea López-González | Moderate the question session. |
| **Facilitator 2** | Dora Yonda | Note-taker. Welcome participants. |
| **General Information** | | |
| **Focus** | Consensus on AUDIT wording | |
| **Discussion time** | 60-90 minutes | |
| **Materials** | 1. Field diary 2. Pen 3. Participant list 4. Cardboard 5. Colored paper 6. Tape 7. Markers | |
| **Phase 1: Idea Generation** | | |
| **What does this phase consist of?** | This question will focus on the naturalness, acceptability, and functionality of the words and illustrations used in the questions, in the standard drink guide, and in the instructions of the AUDIT self-administered and interview versions. Each expert writes or briefly comments on an idea in response to the question. | |
| **Phase 2: Idea Discussion** | | |
| **What does this phase consist of?** | El moderador deja cada idea visible para el grupo. Se realiza una discusión de cada idea aportada por cada experto (se utilizarán preguntas como: ¿hay algún comentario de los integrantes del grupo sobre esta idea?). Se da la oportunidad a los expertos de ajustar las ideas. | |
| **Phase 3: Voting** | | |
| **What does this phase consist of?** | Cada idea final acordada en la fase 2 será marcada con un número y estará visible para todos los participantes. Se realizará la pregunta: ¿A quién le parece que la mejor opción es la (se lee el número de la idea)? Aquellos expertos que estén de acuerdo, deberán levantar la mano. La idea que obtenga 80% o más de acuerdo, será incluida en la adaptación. | |
| **Questions** | | |
| **Round 1 of Questions** | What do you call beverages that contain alcohol? | |
| **Round 2 of Questions** | What do you call a fermented fruit beverage? | |
| **Round 3 of Questions** | What do you do with beverages that contain alcohol? | |
| **Round 4 of Questions** | What do you call the object used to _______ (according to the previous answer) these beverages? | |
| **Round 5 of Questions** | What do you call documents that contain questions and answer options and are handed out to be filled in? | |
| **Round 6 of Questions** | What do you call the act of completing these _______ (according to the previous answer)? | |

Source: own elaboration
