# Supplementary material for: Strengthening alcohol consumption screening in primary care for culturally diverse populations in Latin America: Adaptation and content and face validity of the AUDIT in Guaviare, Colombia
Source: PLoS One. 2026 Sep 30;21(9):e0359625. doi: 10.1371/journal.pone.0359625 (PMC13626350; doi:10.1371/journal.pone.0359625)
Supplement: Supplementary file 5 [file pone.0359625.s004.docx]

**First adapted AUDIT versions**

This appendix presents the first adapted Spanish version of the Alcohol Use Disorders Identification Test (AUDIT), resulting from the cultural adaptation process conducted in Guaviare, Colombia. Both the interviewer and self-administered formats are included. This version constitutes one of the study outcomes and is therefore presented in the language in which it was culturally adapted. An English version is not provided because it was not developed or evaluated as part of this study. For reference, the original English version of the AUDIT is provided in S1 Appendix.

**AUDIT self-administered format: First adapted version (Spanish)**

| **Test de Identificación de Trastornos por consumo de Alcohol (AUDIT): versión autollenado**  **Parte 1** | | | | | | |
| --- | --- | --- | --- | --- | --- | --- |
| **Instrucciones para persona encargada:**  A continuación, va a encontrar un formulario para identificar tempranamente a las personas que toman bebidas alcohólicas. Este formulario tiene dos versiones: una versión de autollenado que solo debe ser llenada por el participante y una versión entrevista que debe ser llenada junto con un personal de salud o agente social o investigador.    El formato que actualmente tiene es la versión autollenado.    **Siga las siguientes instrucciones:**     | | | | | | |
| **Test de Identificación de Trastornos por consumo de Alcohol (AUDIT): versión autollenado**  **Parte 2** | | | | | | |
| **Instrucciones para participante:**    Responda las siguientes 10 preguntas. Le tomarán 10 minutos aproximadamente. Encontrará 6 pasos y 2 cuadros con imágenes para ayudarle a responder las preguntas.     A. Marque con una X la opción que mejor responde a su caso. B. No hay respuestas buenas o malas. C. Solo escriba una respuesta por pregunta. D. Responda con sinceridad. E. Puede pedir más de un formulario en caso de que lo necesite.     Una **bebida alcohólica** hace referencia a cualquier bebida comercial, casera o fermentada que contiene alcohol. Piense las veces que ha tomado alguna de estas bebidas, incluso para bajar la sed o para refrescarse.    **Siga los pasos:** | | | | | | |
| **Paso 1:** escriba una X sobre la imagen de la bebida alcohólica comercial, casera o fermentada que haya tomado en el último año. Cerveza, pola o coronita en botella \|  Cerveza, pola o coronita en lata \|  Aguardiente o guaro \|  Ron \|  Whiskey \|  Tequila;  Champaña \|  Vino \|  Guarapo \|  Chicha \|  Viche \|  Chirrinchi **Paso 2:** responda la siguiente pregunta. | | | | | | |
| **Preguntas** | **Opciones de respuesta** | | | | | **Puntaje** |
| 1. En el último año, ¿con qué frecuencia ha tomado alguna bebida alcohólica? (recuerde los ejemplos de bebidas alcohólicas en el paso 1) | Nunca        **[0 puntos]** | Una o menos veces al mes      **[1 punto]** | 2 o 4 veces al mes      **[2 puntos]** | 2 o 3 veces a la semana      **[3 puntos]** | 4 o más veces a la semana    **[4 puntos]** |  |
| **Paso 3:** Escriba una X sobre las imágenes de los recipientes que use para tomar bebidas alcohólicas comerciales, caseras o fermentadas.  Vaso pequeño \|  Vaso mediano \|  Vaso grande \|  Botella \|  Lata \|;  Copa doble \|  Copa pequeña \|  Copa larga \|  Copa grande \|  Totuma o cuya pequeña \|  Totuma o cuya grande **Paso 4:** A continuación, encontrará una pregunta en donde las opciones de respuestas son sobre el número de tragos, vasos, copas, cuyas o cervezas. Responda la pregunta. | | | | | | |
| **Preguntas** | **Opciones de respuesta** | | | | | **Puntaje** |
| 2. ¿Cuántas copas, vasos, cervezas, tragos o cuyas de bebidas alcohólicas suele tomar en un día de consumo normal? (Recuerde los ejemplos de recipientes en el paso 3) | 1 o 2 copas, vasos, cervezas, tragos o cuyas      **[0 puntos]** | 3 o 4 copas, vasos, cervezas, tragos o cuyas      **[1 punto]** | 5 o 6 copas, vasos, cervezas, tragos o cuyas        **[2 puntos]** | 7, 8 o 9 copas, vasos, cervezas, tragos o cuyas        **[3 puntos]** | 10 o más copas, vasos, cervezas, tragos o cuyas    **[4 puntos]** |  |
| **Paso 5:** A continuación, encontrará preguntas en donde las opciones de respuestas cambian. Esta vez las opciones de respuesta son nunca, menos de una vez al mes, mensualmente, semanalmente, a diario o casi a diario. Responda las siguientes preguntas. | | | | | | |
| **Preguntas** | **Opciones de respuesta** | | | | | **Puntaje** |
| 3. ¿Con qué frecuencia toma 6 o más copas, vasos, cervezas, tragos o cuyas de bebidas alcohólicas en un solo día? | Nunca        **[0 puntos]** | Menos de una vez al mes    **[1 punto]** | Mensualmente        **[2 puntos]** | Semanalmente        **[3 puntos]** | A diario o casi a diario      **[4 puntos]** |  |
| 4. En el último año, ¿Con qué frecuencia le ha sido difícil parar de tomar bebidas alcohólicas? | Nunca        **[0 puntos]** | Menos de una vez al mes    **[1 punto]** | Mensualmente        **[2 puntos]** | Semanalmente        **[3 puntos]** | A diario o casi a diario      **[4 puntos]** |  |
| 5. En el último año, ¿Con qué frecuencia no pudo hacer lo que se esperaba de usted porque había tomado bebidas alcohólicas? | Nunca        **[0 puntos]** | Menos de una vez al mes    **[1 punto]** | Mensualmente        **[2 puntos]** | Semanalmente        **[3 puntos]** | A diario o casi a diario      **[4 puntos]** |  |
| 6. En el último año, ¿Con qué frecuencia ha necesitado tomar alguna bebida alcohólica en ayunas para recuperarse después de haber tomado mucho el día anterior? | Nunca          **[0 puntos]** | Menos de una vez al mes      **[1 punto]** | Mensualmente          **[2 puntos]** | Semanalmente          **[3 puntos]** | A diario o casi a diario        **[4 puntos]** |  |
| 7. En el último año, ¿Con qué frecuencia ha tenido remordimientos o sentimientos de culpa después de haber tomado bebidas alcohólicas? | Nunca        **[0 puntos]** | Menos de una vez al mes    **[1 punto]** | Mensualmente        **[2 puntos]** | Semanalmente        **[3 puntos]** | A diario o casi a diario      **[4 puntos]** |  |
| 8. En el último año, ¿Con qué frecuencia no ha podido recordar lo que sucedió la noche anterior porque había estado tomando bebidas alcohólicas? | Nunca        **[0 puntos]** | Menos de una vez al mes    **[1 punto]** | Mensualmente        **[2 puntos]** | Semanalmente        **[3 puntos]** | A diario o casi a diario      **[4 puntos]** |  |
| **Paso 6:** A continuación, encontrará preguntas en donde las opciones de respuestas cambian. Esta vez solo encontrará tres opciones de respuesta. Responda las siguientes preguntas. | | | | | | |
| **Preguntas** | **Opciones de respuesta** | | | | | **Puntaje** |
| 9. ¿Usted o alguna otra persona ha resultado herido porque usted había tomado alguna bebida alcohólica? | No        **[0 puntos]** |  | Sí, pero no en el curso del último año    **[2 puntos]** |  | Sí, el último año      **[4 puntos]** |  |
| 10. ¿Algún familiar, amigo, médico o personal de la salud ha mostrado preocupación por su forma de tomar bebidas alcohólicas o le han sugerido que deje de tomar? | No          **[0 puntos]** |  | Sí, pero no en el curso del último año      **[2 puntos]** |  | Sí, el último año        **[4 puntos]** |  |
| **Total de puntos** | | | | | |  |
| Muchas gracias por responder todas las preguntas.  Entregue este formulario a la persona encargada. | | | | | | |

**Source:** Babor TF, Higgins-Biddle JC, Saunders JB, Monteiro MG. AUDIT formulario de identificación de los transtornos debidos al consumo de alcohol [Internet]. Organización Mundial de la Salud. Ginebra, Suiza; 2001 [citado el 14 de febrero de 2023]. Available at: <https://www.paho.org/hq/dmdocuments/2009/AUDIT_spa.pdf.>

**AUDIT interview format: First adapted version (Spanish)**

**Test de Identificación de Trastornos por consumo de Alcohol (AUDIT): versión entrevista**

**Parte 1**

**Instrucciones para persona encargada:**

A continuación, va a encontrar un formulario para identificar tempranamente a las personas que toman bebidas alcohólicas. Este formulario tiene dos versiones: una versión de autollenado que solo debe ser llenada por el participante y una versión entrevista que debe ser llenada junto con un personal de salud o agente social o investigador.

El formato que actualmente tiene es la versión entrevista.

**Siga las siguientes instrucciones:**

**Test de Identificación de Trastornos por consumo de Alcohol (AUDIT): versión entrevista**

**Parte 2**

**Lea al participante lo siguiente:**

A continuación, le haré un formulario con 10 preguntas. Le tomarán 10 minutos aproximadamente. Vamos a seguir 8 pasos y le mostraré 2 cuadros con imágenes para ayudarle a responder las preguntas. No hay respuestas buenas o malas. Responda con sinceridad.

Una **bebida alcohólica** hace referencia a cualquier bebida comercial, casera o fermentada que contiene alcohol. Piense las veces que ha tomado alguna de estas bebidas, incluso para bajar la sed o para refrescarse.

**Siga los pasos:**

**Paso 1:** muestre las siguientes imágenes al participante y pregunte: ¿Cuál de las siguientes bebidas alcohólicas comerciales, caseras o fermentadas ha tomado en el último año? Marque una X en la respuesta del participante.

| Cerveza, pola o coronita en botella | Cerveza, pola o coronita en lata |     Aguardiente o guaro |     Ron |     Whiskey |     Tequila |
| --- | --- | --- | --- | --- | --- |
|   Champaña |   Vino |   Guarapo |   Chicha |   Viche |   Chirrinchi |

**Paso 2:** lea la siguiente pregunta.

1. En el último año, ¿con qué frecuencia ha tomado alguna bebida alcohólica? (recuérdele los ejemplos de bebidas alcohólicas en el paso 1)

a. Nunca (0 puntos)
b. Una o menos veces al mes (1 punto)
c. 2 o 4 veces al mes (2 puntos)
d. 2 o 3 veces a la semana (3 puntos)
e. 4 o más veces a la semana (4 puntos)

**Paso 3:** si la respuesta del participante fue “Nunca” en la pregunta 1, continúe a las preguntas 9 y 10.

**Paso 4:** muestre las siguientes imágenes al participante y pregunte: ¿Cuál de los siguientes recipientes usa para tomar bebidas alcohólicas comerciales, caseras o fermentadas? Marque una X en la respuesta del participante.

|   Vaso pequeño |   Vaso mediano |   Vaso grande |   Botella |   Lata |  |
| --- | --- | --- | --- | --- | --- |
|     Copa doble |     Copa pequeña |   Copa larga |   Copa grande |     Totuma o cuya pequeña |     Totuma o cuya grande |

**Paso 5:** a continuación, encontrará una pregunta en donde las opciones de respuestas son sobre el número de tragos, vasos, copas, cuyas o cervezas. Realice la siguiente pregunta.

2. ¿Cuántas copas, vasos, cervezas, tragos o cuyas de bebidas alcohólicas suele tomar en un día de consumo normal? (Recuérdele los ejemplos de recipientes en el paso 3)

a. 1 o 2 (0 puntos)
b. 3 o 4 (1 punto)
c. 5 o 6 (2 puntos)
d. 7, 8 o 9 (3 puntos)
e. 10 o más (4 puntos)

**Paso 6:** a continuación, encontrará preguntas en donde las opciones de respuestas cambian. Esta vez las opciones de respuesta son nunca, menos de una vez al mes, mensualmente, semanalmente, a diario o casi a diario. Realice las siguientes preguntas.

3. ¿Con qué frecuencia toma 6 o más copas, vasos, cervezas, tragos o cuyas de bebidas alcohólicas en un solo día?

a. Nunca (0 puntos)
b. Menos de una vez al mes (1 punto)
c. Mensualmente (2 puntos)
d. Semanalmente (3 puntos)
e. A diario o casi a diario (4 puntos)

**Paso 7:** sume el puntaje de las preguntas 2 y 3.

Si esta suma es igual a 0 puntos, continúe a la pregunta 9 y 10.

Si esta suma es igual o mayor a 1 punto, continúe con la pregunta 4.

4. En el último año, ¿Con qué frecuencia le ha sido difícil parar de tomar bebidas alcohólicas?

a. Nunca (0 puntos)
b. Menos de una vez al mes (1 punto)
c. Mensualmente (2 puntos)
d. Semanalmente (3 puntos)
e. A diario o casi a diario (4 puntos)

5. En el último año, ¿Con qué frecuencia no pudo hacer lo que se esperaba de usted porque había tomado bebidas alcohólicas?

a. Nunca (0 puntos)
b. Menos de una vez al mes (1 punto)
c. Mensualmente (2 puntos)
d. Semanalmente (3 puntos)
e. A diario o casi a diario (4 puntos)

6. En el último año, ¿Con qué frecuencia ha necesitado tomar alguna bebida alcohólica en ayunas para recuperarse después de haber tomado mucho el día anterior?

a. Nunca (0 puntos)
b. Menos de una vez al mes (1 punto)
c. Mensualmente (2 puntos)
d. Semanalmente (3 puntos)
e. A diario o casi a diario (4 puntos)

7. En el último año, ¿Con qué frecuencia ha tenido remordimientos o sentimientos de culpa después de haber tomado bebidas alcohólicas?

a. Nunca (0 puntos)
b. Menos de una vez al mes (1 punto)
c. Mensualmente (2 puntos)
d. Semanalmente (3 puntos)
e. A diario o casi a diario (4 puntos)

8. En el último año, ¿Con qué frecuencia no ha podido recordar lo que sucedió la noche anterior porque había estado tomando bebidas alcohólicas?

a. Nunca (0 puntos)
b. Menos de una vez al mes (1 punto)
c. Mensualmente (2 puntos)
d. Semanalmente (3 puntos)
e. A diario o casi a diario (4 puntos)

**Paso 8:** a continuación, encontrará preguntas en donde las opciones de respuestas cambian. Esta vez solo encontrará tres opciones de respuesta. Realice las siguientes preguntas.

9. ¿Usted o alguna otra persona ha resultado herido porque usted había tomado alguna bebida alcohólica?

a. No (0 puntos)
b. Sí, pero no en el curso del último año (2 puntos)
c. Sí, en el último año (4 puntos

10. ¿Algún familiar, amigo, médico o personal de la salud ha mostrado preocupación por su forma de tomar bebidas alcohólicas o le han sugerido que deje de tomar?

a. No (0 puntos)
b. Sí, pero no en el curso del último año (2 puntos)
c. Sí, en el último año (4 puntos)

**Sume los puntos obtenidos por el participante en cada pregunta y regístrelo acá: ____________**

**Source:** Babor TF, Higgins-Biddle JC, Saunders JB, Monteiro MG. AUDIT formulario de identificación de los transtornos debidos al consumo de alcohol [Internet]. Organización Mundial de la Salud. Ginebra, Suiza; 2001 [citado el 14 de febrero de 2023]. Available at: <https://www.paho.org/hq/dmdocuments/2009/AUDIT_spa.pdf.>
