# Supplementary material for: Strengthening alcohol consumption screening in primary care for culturally diverse populations in Latin America: Adaptation and content and face validity of the AUDIT in Guaviare, Colombia
Source: PLoS One. 2026 Sep 30;21(9):e0359625. doi: 10.1371/journal.pone.0359625 (PMC13626350; doi:10.1371/journal.pone.0359625)
Supplement: Supplementary file 6 [file pone.0359625.s005.docx]

**Cognitive Interview Guide**

| **Project:** Cultural adaptation and psychometric validation of the AUDIT scale for screening hazardous alcohol consumption in an adult population in Guaviare.  **Cognitive Interview Guide**  **Field Test** | | |
| --- | --- | --- |
| **Team** | | |
|  | *Names* | *Roles* |
| **Facilitator 1** | Andrea López-González | Moderate the question session. |
| **General Information** | | |
| **Discussion time** | 45-60 minutes | |
| **Materials** | a. Field diary b. Pen c. Interview guide d. Voice recorder e. AUDIT interview or self-administered format (first adapted version) | |
| **Questions** | | |
| **Main Questions** | **Probing Questions** | |
| What did you understand from the instructions? | Should anything be changed in the instructions? Why? | |
| What did you understand by the question...? | What do you think you are being asked?  What did you have to think about to answer the question?  Did you have any difficulty answering the question? Why? | |
| What did you understand by the answer option...? | Explain in your own words what this answer option means...  How did you arrive at that answer?  Was it easy or difficult to choose an answer? Why? | |
| What did you understand by the word...? |  | |
| What did you understand by the expression...? |  | |

Source: own elaboration
