# Supplementary material for: Strengthening alcohol consumption screening in primary care for culturally diverse populations in Latin America: Adaptation and content and face validity of the AUDIT in Guaviare, Colombia
Source: PLoS One. 2026 Sep 30;21(9):e0359625. doi: 10.1371/journal.pone.0359625 (PMC13626350; doi:10.1371/journal.pone.0359625)
Supplement: Supplementary file 7 [file pone.0359625.s007.docx]

Inclusivity in global research

PLOS’ policy on inclusivity in global research aims to improve transparency in the reporting of research performed outside of researchers’ own country or community and ensures that PLOS publications reporting global research adhere to high standards for research ethics and authorship. Authors of relevant research articles may be asked to complete the questionnaire below, which outlines ethical, cultural, and scientific considerations specific to inclusivity in global research. This questionnaire may be requested when researchers have travelled to a different country to conduct research, if research uses samples collected in another country, research with Indigenous populations or their lands, or if research is on cultural artefacts. Researchers travelling to another country solely to use laboratory equipment will not normally be required to complete the questionnaire. However, the questionnaire can be requested at the journal’s discretion for any submission – if you have been requested to complete this questionnaire by the PLOS journal you submitted to, please do so.

Please complete the questionnaire below and include this as a Supporting Information file with your manuscript. Note that if your paper is accepted for publication, this checklist will be published with your article in the supporting information files. Please ensure that you reference the checklist in the main body of your manuscript. We suggest adding a subsection ‘Inclusivity in global research’ to your Methods section and adding the following sentence: “Additional information regarding the ethical, cultural, and scientific considerations specific to inclusivity in global research is included in the Supporting Information (SX Checklist)”

The questions have been designed to be applicable to a wide range of study types, and there are subsections for both human subjects research and non-human subjects research. If any of the questions are not relevant to your research please mark them as “N/A” as appropriate.

**Ethical considerations, permits and authorship**

*This section is applicable to all research types.*

Provide details as to who granted permissions and/or consent for the study to take place in the Methods section of your manuscript. This should include the names of **all** ethics boards, governmental organizations, community leaders or other bodies that provided approval for the study. If individuals provided approval refer to these people by their role or title but do not list their name(s).

Reported on page number: Pages 11-12

If there were any deviations from the study protocol after approval was obtained please provide details of these changes in the Methods section of your manuscript.
Did this study involve local collaborators that are residents of the country where the research was conducted or members of the community studied? If you do not have any authors from said communities, please provide an explanation for this below.

Reported on page number: N/A

Yes. The study was conducted by Colombian researchers in collaboration with local stakeholders in Guaviare. Community members were actively engaged throughout the cultural adaptation process through nominal group sessions and cognitive interviews, and one of the co-authors is a member of the study community.

Everyone listed as an author should meet PLOS’ criteria for authorship and all individuals who meet these criteria should be included in the author byline, rather than the acknowledgements. For further information please see the journal’s Authorship Policy.

**Human subjects research (e.g. health research, medical research, cross-cultural psychology)**

Did you obtain written informed consent from a representative of the local community or region before the research took place? How did you establish who speaks for the community? Details of written informed consent obtained from study participants should be reported separately in the Methods section of your manuscript.

No. Written informed consent was not obtained from a representative of the local community prior to the study. The study was approved by the Ethics Committee of Hospital Universitario San Ignacio and Pontificia Universidad Javeriana (Minutes No. 04/2024; March 7, 2024). Before implementation, the study was presented to the Secretaría de Salud del Guaviare (Guaviare Department Health Secretariat), Colombia, and discussed with community leaders from Charras-Boquerón. Community representatives were identified through the established community leadership structure, and these recognized leaders were consulted before the study began to ensure that the research addressed local priorities and was appropriate for the community. One of the co-authors, who is a member of the study community, participated in these discussions and throughout the research process, helping ensure that community perspectives informed the cultural adaptation process. Written informed consent was obtained separately from all study participants before enrollment, as described in the Methods section.

How did members of the local community provide input on the aims of the research investigation, its methodology, and its anticipated outcome(s)?

Community members did not co-develop the research aims or study design. However, they played a central role in the cultural adaptation process, which was a key component of the study methodology. Community input was obtained through nominal group sessions and cognitive interviews, during which participants identified culturally relevant terminology, highlighted barriers to comprehension, and provided recommendations to improve the clarity, acceptability, and cultural appropriateness of the AUDIT. Their feedback directly informed the revisions made during both phases of the adaptation process and the development of the final adapted versions of the interviewer-administered and self-administered AUDIT. In addition, one of the co-authors, who is a member of the study community, participated in reviewing and discussing the study findings, helping ensure that the interpretation of the results reflected the local context and community perspectives.

When engaging with the local community, how did you ensure that the informed consent documents and other materials could be understood by local stakeholders?

All study materials, including the informed consent form, were developed in Spanish, the native language of the participants. Before data collection, the informed consent form was pilot-tested with three community members from Guaviare. No modifications were required following this process. Throughout the study, one of the co-authors, who is a member of the study community, participated in the data collection process and helped ensure that communication with participants was culturally appropriate and easily understood.

Will the findings of the research be made available in an understandable format to stakeholders in the community where the study was conducted (e.g. via a presentation, summary report, copies of publications, etc.)? Please provide details of how this will be achieved.

Yes. As part of a broader community engagement and social appropriation of knowledge strategy, the research team disseminated the study findings through several channels. Results were shared directly with community leaders and members of a lived-experience advisory panel. A training session on screening for hazardous alcohol consumption was also delivered to staff from the Departmental Health Secretariat (Secretaría Departamental de Salud del Guaviare) and primary care center personnel in Guaviare. In addition, an audio program for the community, called "Tejiendo Salud" ("Weaving Health"), is currently being developed to share the study results through social media in an accessible format for the general population.

**Non-human subjects research using specimens/ animals collected as part of the study, or those housed in archival collections. Examples include archaeology, paleontology, botany and zoology.**

Did the permission you obtained from a local authority to perform the study include an agreement on access to outputs and benefit sharing? This may include procedures to enable fair distribution of the benefits and resources arising from the research performed. Please include any details of Prior Informed Consent and Benefit Sharing Agreements obtained. These may be required by field-specific regulations, for example the Convention on Biological Diversity (CBD) and the associated Nagoya Protocol.

N/A

If the material used in your study was imported, please A) provide the year it was imported and B) indicate whether permits were obtained to import/export the materials used, C) provide details of any permits obtained. If this information is not available, please indicate this.

N/A

If you used archival specimens, please state how the material used in your study was acquired by the institute it is held in and provide details of any permits obtained for the original excavations/ sample collection. If this information is not available, please indicate this.

N/A

How was the potential cultural significance of the materials collected in your study to local communities considered in your research design? Were Indigenous peoples and/or local researchers and institutions involved with archaeological excavations / collection of specimens? If so, please provide a description of their involvement.

N/A

If your manuscript includes photographs of human remains please indicate whether authors obtained permission from descendants or affiliated cultural communities to do so.

N/A
