# Supplementary material for: Strengthening alcohol consumption screening in primary care for culturally diverse populations in Latin America: Adaptation and content and face validity of the AUDIT in Guaviare, Colombia
Source: PLoS One. 2026 Sep 30;21(9):e0359625. doi: 10.1371/journal.pone.0359625 (PMC13626350; doi:10.1371/journal.pone.0359625)
Supplement: Supplementary file 8 [file pone.0359625.s010.docx]

**Response to Reviewers**

**Manuscript Title:** Strengthening alcohol consumption screening in primary care for culturally diverse populations in Latin America: adaptation and content and face validity of the AUDIT in Guaviare, Colombia

**Manuscript ID:** PONE-D-25-64964

Dear Editor and Reviewers,

We thank the academic editor and reviewers for their careful reading of our manuscript and for their constructive comments, which have substantially strengthened the paper. Below we provide a point-by-point response to each comment.

| **Reviewer/Editor Comment** | **Response** |
| --- | --- |
| **Journal Requirements** | |
| If applicable, we recommend that you deposit your laboratory protocols in protocols.io to enhance the reproducibility of your results. | This comment does not apply to our study, as it did not involve laboratory protocols. |
| PLOS supports the use of ORCID, and we encourage all coauthors to register for an ORCID iD and use it as well. | Thank you for this reminder. All coauthors will verify their ORCID iDs within the submission system. |
| 1. Please ensure that your manuscript meets PLOS ONE's style requirements, including those for file naming. | We carefully revised the manuscript and supporting files to ensure compliance with the PLOS ONE formatting and style requirements, including file naming, figure and table formatting, and supporting information. |
| 2. Please include a complete copy of PLOS' questionnaire on inclusivity in global research in your revised manuscript. | Thank you for this reminder. A completed copy of the questionnaire on inclusivity in global research has been uploaded as Supporting Information. |
| 3. Please expand the acronym “NIHR” (as indicated in your financial disclosure) so that it states the name of your funders in full. | Thank you for pointing this out. The acronym “NIHR” has been expanded to state the full name of the funder in the cover letter, for the editorial team to update the online submission form accordingly. |
| 4. Your ethics statement should only appear in the Methods section of your manuscript. | Thank you for this observation. The ethics statement has been moved to the Methods section and removed from all other sections of the manuscript. |
| 5. Thank you for providing your underlying data as Supporting Information. We note that the data set contains text or data that is not in English. | Thank you for this comment. We appreciate the opportunity to clarify this point. We have translated into English and re-uploaded the supporting materials that are not study outcomes, including S1 Appendix (which now includes both the original English and Spanish versions of the AUDIT), S2 Appendix (Structured Observation Guide), S3 Appendix (Nominal Group Technique Guide), and S5 Appendix (Cognitive Interview Guide).  S4 and S6 Appendices, however, present the first adapted and final adapted Spanish versions of the AUDIT (both interviewer-administered and self-administered formats), which constitute the primary outcomes of this cultural adaptation study. These instruments were culturally adapted and evaluated in Spanish in Guaviare, Colombia. Translating them into English would create versions that were neither developed nor evaluated as part of this study and therefore would not accurately represent the study outcomes.  To facilitate interpretation by international readers, we have included the original English version of the AUDIT in S1 Appendix and added explanatory notes to S4 and S6 Appendices clarifying why these instruments are presented only in Spanish. We respectfully request that these appendices be retained in Spanish, as they represent the validated outputs of the cultural adaptation process. |
| 6. We note [S6_Appendix.pdf and S4_Appendix.docx] in your submission contain copyrighted images. | Thank you for this comment. We have obtained written permission from the original copyright holder to publish the image(s) in [S6_Appendix.pdf and S4_Appendix.docx] under the CC BY 4.0 license, as required. The signed Content Permission Form is attached to this submission for your review. |
| 7. If the reviewer comments include a recommendation to cite specific previously published works, please review and evaluate these publications to determine whether they are relevant and should be cited. | No reviewer recommended citing specific previously published works in their comments. Therefore, no additional citations were considered or added in response to this point. |
| **Reviewer Comments** | |
| In a study of this nature, the data should be completely anonymized, which does not seem to be the case. Greater anonymization of the data is recommended, especially that in Table 1, page 10. | Thank you for this important comment. We have further anonymized the data in Table 1 and the accompanying dataset to ensure participant confidentiality.  Specifically, participants' age was recoded into three-year age ranges (18–29, 30–44, 45–65) instead of exact ages. Educational level was recoded into three broad categories (low, middle, high) instead of specific educational attainment levels. Social group categories with very small cell sizes (migrant and peace agreement signatory) were combined into a single "Other" category to prevent indirect identification of participants belonging to these small subgroups.  In addition, we anonymized the underlying dataset by recoding sex, place of origin, and place of residence into numerical codes rather than identifiable text values. No names, contact information, exact dates, or specific location data are included in the shared dataset.  We believe these changes substantially reduce the risk of participant identification, in accordance with the journal's data-sharing requirements, while preserving the scientific value of the sociodemographic information presented. |
| The Conclusion seems to repeat the sentences from the Discussion and needs improvement. | Thank you for this observation. The Conclusion has been rewritten to synthesize the study's key contributions rather than repeat content already presented in the Discussion. |
| The manuscript lacks a section clearly identifying the study limitations. | Thank you for this observation. A dedicated Limitations section has been added to the manuscript. |
| A clearer distinction between the terms “translation” and “cultural adaptation” could be included, explicitly stating that cultural adaptation goes far beyond linguistic translation (this being a significant and original aspect of the study). | We thank the reviewer for this valuable suggestion. We have expanded the Introduction to explicitly distinguish between translation and cultural adaptation, clarifying that cultural adaptation extends beyond linguistic translation to ensure semantic, content, and technical equivalence. |
| It would also be desirable to include a reference to the work of Willis (2005) on cognitive interviewing. | Thank you for this recommendation. A reference to Willis (2005) on cognitive interviewing has been added to the Introduction. |
| Furthermore, it seems useful to provide a clearer clarification of the concepts of face validity and content validity for readers who may be less familiar with psychometrics. | Thank you for this suggestion. A clarification of the concepts of content validity and face validity has been added to the Introduction. |
| It would be important to specify the cognitive interview procedures, indicating which cognitive model was used and possibly presenting examples of cognitive probing questions. | Thank you for this comment. We have specified the cognitive interviewing model used (Miller et al., 2014) and included examples of cognitive probing questions in the Methods section (Phase 2). |
| The bibliography concerning the AUDIT instrument is very consistent and includes key reference works. However, Wild et al. (2005) and Beaton et al. (2000) could also be included regarding cultural issues and test adaptation. | Thank you for this suggestion. Both references have been incorporated into the Introduction in relation to the process of translation and cultural adaptation of instruments. |
| The manuscript identifies interpretation difficulties associated with the specific cultural context, as well as differences between the interview-administered and self-administered formats. Particular attention should be given to Table 5, which contains highly significant information and demonstrates that the study did not merely consist of a linguistic adaptation. The table would be even more comprehensive with the inclusion of a fifth column entitled “Justification for Adaptation”, where observations could be added regarding, for example, semantic confusion identified during interviews or improved comprehension among participants with low educational attainment. This addition would clarify the reasons underlying the proposed modifications. | We thank the reviewer for this valuable suggestion. A fifth column, “Justification for Adaptation,” has been added to the table (now Table 4), categorizing each modification as Semantic Clarity, Comprehension and Readability, or Content Relevance, with a corresponding table footnote defining each category. |
| We also suggest placing particular emphasis on the fact that “guarapo” is not considered an alcoholic beverage within that cultural context, which constitutes a highly relevant theoretical issue and an innovative finding. This cultural invisibility of alcohol consumption should be highlighted in both the discussion and conclusions. | Thank you for highlighting this important point. The cultural invisibility of “guarapo” as an alcoholic beverage has been emphasized in both the Discussion and Conclusion sections. |
| Finally, the manuscript lacks a dedicated section on study limitations (an important aspect), such as justification for the number of participants (possibly based on information saturation or interpretative redundancy), potential social desirability bias, limited cultural diversity with all participants being Spanish-speaking, or the absence of quantitative psychometric validation | Thank you for this detailed feedback. The Limitations section now addresses the justification for the number of participants, potential social desirability bias, the limited cultural diversity of the sample (all Spanish-speaking participants), and the absence of quantitative psychometric validation. |
| As previously suggested, we recommend reinforcing aspects related to the fact that the study goes beyond the adaptive translation of an instrument, which carries important methodological implications for this type of research. Likewise, the invisibility of a particular form of alcohol consumption (in the case of “guarapo”) should be emphasised, given its implications for understanding the notion of culturally embedded alcohol consumption. | We thank the reviewer for this valuable suggestion. We have reinforced the distinction between translation and cultural adaptation throughout the manuscript, and explicitly connected this distinction to the case of “guarapo” as evidence that the study extends beyond linguistic translation. These adjustments have been incorporated in the Introduction, Discussion, Conclusion, and Table 4. |
| We suggest the possible inclusion of the authors previously mentioned in the methodological appraisal. | Thank you for this suggestion. The initials of the authors who participated in the cultural adaptation process have been included in the Methods section. |
| It is also important to review the references, as references numbers 4, 7, 20, and 21 appear to be incomplete. | Thank you for this observation. All references were reviewed, and references 4, 7, 20, and 21 have been completed. |

Sincerely,

Andrea López-González

On behalf of all co-authors

Department of Clinical Epidemiology and Biostatistics, School of Medicine, Pontificia Universidad Javeriana, Bogotá, Colombia
