# Supplementary material for: Strengthening alcohol consumption screening in primary care for culturally diverse populations in Latin America: Adaptation and content and face validity of the AUDIT in Guaviare, Colombia
Source: PLoS One. 2026 Sep 30;21(9):e0359625. doi: 10.1371/journal.pone.0359625 (PMC13626350; doi:10.1371/journal.pone.0359625)
Supplement: Peer review history

# Peer review: Decision Letter 0

**DOI:** 10.1371/journal.pone.0359625.r001

**Authors:** Maria Nogueira

3 Jun 2026

Dear Dr. López-González,

Thank you for submitting your manuscript to PLOS ONE. After careful consideration, we feel that it has merit but does not fully meet PLOS ONE’s publication criteria as it currently stands. Therefore, we invite you to submit a revised version of the manuscript that addresses the points raised during the review process.

Please submit your revised manuscript by Jul 18 2026 11:59PM. If you will need more time than this to complete your revisions, please reply to this message or contact the journal office at plosone@plos.org. When you're ready to submit your revision, log on to https://www.editorialmanager.com/pone/ and select the 'Submissions Needing Revision' folder to locate your manuscript file.

- A letter that responds to each point raised by the academic editor and reviewer(s). You should upload this letter as a separate file labeled 'Response to Reviewers'.
- A marked-up copy of your manuscript that highlights changes made to the original version. You should upload this as a separate file labeled 'Revised Manuscript with Track Changes'.
- An unmarked version of your revised paper without tracked changes. You should upload this as a separate file labeled 'Manuscript'.

If you would like to make changes to your financial disclosure, please include your updated statement in your cover letter. Guidelines for resubmitting your figure files are available below the reviewer comments at the end of this letter.

If applicable, we recommend that you deposit your laboratory protocols in protocols.io to enhance the reproducibility of your results. Protocols.io assigns your protocol its own identifier (DOI) so that it can be cited independently in the future. For instructions see: https://journals.plos.org/plosone/s/submission-guidelines#loc-laboratory-protocols. Additionally, PLOS ONE offers an option for publishing peer-reviewed Lab Protocol articles, which describe protocols hosted on protocols.io. Read more information on sharing protocols at https://plos.org/protocols?utm_medium=editorial-email&utm_source=authorletters&utm_campaign=protocols.

As the corresponding author, your ORCID iD is verified in the submission system and will appear in the published article. PLOS supports the use of ORCID, and we encourage all coauthors to register for an ORCID iD and use it as well. Please encourage your coauthors to verify their ORCID iD within the submission system before final acceptance, as unverified ORCID iDs will not appear in the published article. *Only*  the individual author can complete the verification step; PLOS staff *cannot*  verify ORCID iDs on behalf of authors.

We look forward to receiving your revised manuscript.

Kind regards,

Maria José Nogueira, Ph.D.

Academic Editor

PLOS One

**Journal Requirements:**

When submitting your revision, we need you to address these additional requirements.

1. Please ensure that your manuscript meets PLOS ONE's style requirements, including those for file naming. The PLOS ONE style templates can be found at

https://journals.plos.org/plosone/s/file?id=wjVg/PLOSOne_formatting_sample_main_body.pdf and

https://journals.plos.org/plosone/s/file?id=ba62/PLOSOne_formatting_sample_title_authors_affiliations.pdf

2. Please include a complete copy of PLOS’ questionnaire on inclusivity in global research in your revised manuscript. Our policy for research in this area aims to improve transparency in the reporting of research performed outside of researchers’ own country or community. The policy applies to researchers who have travelled to a different country to conduct research, research with Indigenous populations or their lands, and research on cultural artefacts. The questionnaire can also be requested at the journal’s discretion for any other submissions, even if these conditions are not met.  Please find more information on the policy and a link to download a blank copy of the questionnaire here: https://journals.plos.org/plosone/s/best-practices-in-research-reporting. Please upload a completed version of your questionnaire as Supporting Information when you resubmit your manuscript

3. Please expand the acronym “NIHR” (as indicated in your financial disclosure) so that it states the name of your funders in full.

This information should be included in your cover letter; we will change the online submission form on your behalf.

4. Your ethics statement should only appear in the Methods section of your manuscript. If your ethics statement is written in any section besides the Methods, please move it to the Methods section and delete it from any other section. Please ensure that your ethics statement is included in your manuscript, as the ethics statement entered into the online submission form will not be published alongside your manuscript.

5. Thank you for providing your underlying data as Supporting Information.

We note that the data set contains text or data that is not in English. Please note that PLOS is an English-language publisher, so we require data sets to be provided in English as well. Please upload an English-language version of your data set.

This will also allow us to determine if your data follows PLOS standards per our Data Availability policy here: https://journals.plos.org/plosone/s/data-availability

6. We note [ S6_Appendix.pdf ] in your submission contain copyrighted images. All PLOS content is published under the Creative Commons Attribution License (CC BY 4.0), which means that the manuscript, images, and Supporting Information files will be freely available online, and any third party is permitted to access, download, copy, distribute, and use these materials in any way, even commercially, with proper attribution. For more information, see our copyright guidelines: http://journals.plos.org/plosone/s/licenses-and-copyright.

We require you to either (1) present written permission from the copyright holder to publish these figures specifically under the CC BY 4.0 license, or (2) remove the figures from your submission:

1. You may seek permission from the original copyright holder of Figure [ S6_Appendix.pdf ] to publish the content specifically under the CC BY 4.0 license.

We recommend that you contact the original copyright holder with the Content Permission Form (http://journals.plos.org/plosone/s/file?id=7c09/content-permission-form.pdf) and the following text:

“I request permission for the open-access journal PLOS ONE to publish XXX under the Creative Commons Attribution License (CCAL) CC BY 4.0 (http://creativecommons.org/licenses/by/4.0/). Please be aware that this license allows unrestricted use and distribution, even commercially, by third parties. Please reply and provide explicit written permission to publish XXX under a CC BY license and complete the attached form.”

Please upload the completed Content Permission Form or other proof of granted permissions as an "Other" file with your submission.

In the figure caption of the copyrighted figure, please include the following text: “Reprinted from [ref] under a CC BY license, with permission from [name of publisher], original copyright [original copyright year].”

2. If you are unable to obtain permission from the original copyright holder to publish these figures under the CC BY 4.0 license or if the copyright holder’s requirements are incompatible with the CC BY 4.0 license, please either i) remove the figure or ii) supply a replacement figure that complies with the CC BY 4.0 license. Please check copyright information on all replacement figures and update the figure caption with source information. If applicable, please specify in the figure caption text when a figure is similar but not identical to the original image and is therefore for illustrative purposes only.

7. If the reviewer comments include a recommendation to cite specific previously published works, please review and evaluate these publications to determine whether they are relevant and should be cited. There is no requirement to cite these works unless the editor has indicated otherwise.

**Additional Editor Comments:**

The article has been analyzed and revised, and the authors should carefully consider all the suggestions and considerations raised.

[Note: HTML markup is below. Please do not edit.]

Reviewers' comments:

Reviewer's Responses to Questions

**Comments to the Author**

1. Is the manuscript technically sound, and do the data support the conclusions?

Reviewer #1: Yes

Reviewer #2: Yes

**********

2. Has the statistical analysis been performed appropriately and rigorously?

Reviewer #1: Yes

Reviewer #2: Yes

**********

3. Have the authors made all data underlying the findings in their manuscript fully available?

The PLOS Data policy

Reviewer #1: Yes

Reviewer #2: Yes

**********

4. Is the manuscript presented in an intelligible fashion and written in standard English?

Reviewer #1: Yes

Reviewer #2: Yes

**********

Reviewer #1: In a study of this nature, the data should be completely anonymized, which does not seem to be the case. Greater anonymization of the data is recommended, especially that in Table 1, page 10.

The Conclusion seems to repeat the sentences from the Discussion and needs improvement.

Reviewer #2: General Considerations Regarding the Article

The topic is highly important and original within the fields of public health, the epidemiology of alcoholism, cultural adaptation of assessment instruments, and qualitative methodology. The literature review is very well structured and presented; the study addresses a concrete problem—the need for the cultural adaptation of an assessment instrument—and outlines a set of robust qualitative methodological procedures, following a logical, coherent, and clear sequence. Data presentation is highly comprehensive; the discussion is well articulated with both the research problem and the literature; however, the manuscript lacks a section clearly identifying the study limitations. The bibliographic references are appropriate to the various themes addressed in the article.

Below, we present an appraisal of each of the structural components of the work, along with some suggestions for improvement.

Theoretical Framework

The thematic review is very well organised, addressing aspects such as the importance of alcohol consumption and the AUDIT instrument, the need for cultural adaptation, and the characterisation of the context. There is a very clear articulation with the study objectives, as well as strong institutional grounding (WHO, ITC, Ministry of Health, among others).

A clearer distinction between the terms “translation” and “cultural adaptation” could be included, explicitly stating that cultural adaptation goes far beyond linguistic translation (this being a significant and original aspect of the study). It would also be desirable to include a reference to the work of Willis (2005) on cognitive interviewing. Furthermore, it seems useful to provide a clearer clarification of the concepts of face validity and content validity for readers who may be less familiar with psychometrics.

Methodology

The study design and methodology demonstrate considerable robustness. There is good articulation between the sociocultural context, public health, and qualitative methodology. The fact that the researchers belong to different disciplinary fields certainly contributed to a more appropriate methodological approach and, consequently, to a better understanding of the phenomenon under study.

Particularly noteworthy are the inclusion of cognitive interviews, the collaboration between researchers and the community, the heterogeneity of the purposive sampling strategy, and the use of both interview-administered and self-administered formats. The use of structured observation and the nominal group technique should also be highlighted, as these elements collectively introduce methodological rigour and detail.

It would be important to specify the cognitive interview procedures, indicating which cognitive model was used and possibly presenting examples of cognitive probing questions. The bibliography concerning the AUDIT instrument is very consistent and includes key reference works. However, Wild et al. (2005) and Beaton et al. (2000) could also be included regarding cultural issues and test adaptation.

Results

The results are extensively detailed, with references to participants’ discourse and “speech acts”. The manuscript identifies interpretation difficulties associated with the specific cultural context, as well as differences between the interview-administered and self-administered formats.

Particular attention should be given to Table 5, which contains highly significant information and demonstrates that the study did not merely consist of a linguistic adaptation. The table would be even more comprehensive with the inclusion of a fifth column entitled “Justification for Adaptation”, where observations could be added regarding, for example, semantic confusion identified during interviews or improved comprehension among participants with low educational attainment. This addition would clarify the reasons underlying the proposed modifications.

We also suggest placing particular emphasis on the fact that “guarapo” is not considered an alcoholic beverage within that cultural context, which constitutes a highly relevant theoretical issue and an innovative finding. This cultural invisibility of alcohol consumption should be highlighted in both the discussion and conclusions.

Finally, the manuscript lacks a dedicated section on study limitations (an important aspect), such as justification for the number of participants (possibly based on information saturation or interpretative redundancy), potential social desirability bias, limited cultural diversity with all participants being Spanish-speaking, or the absence of quantitative psychometric validation.

Discussion and Conclusion

This section is very well developed, providing a clarifying articulation between the scientific literature, the results obtained, the clinical relevance of the study, and the sociocultural contextualisation. Topics addressed include the issue of standard drink units, the concept of traditional beverages, and the universality of the concept of the “standard drink”, which here reveals important cultural limitations.

As previously suggested, we recommend reinforcing aspects related to the fact that the study goes beyond the adaptive translation of an instrument, which carries important methodological implications for this type of research. Likewise, the invisibility of a particular form of alcohol consumption (in the case of “guarapo”) should be emphasised, given its implications for understanding the notion of culturally embedded alcohol consumption.

References

The references, comprising 33 entries, adequately represent the range of themes addressed and provide strong theoretical and methodological support. The bibliography includes international references encompassing normative and organisational dimensions, cultural particularities, clinical and epidemiological aspects, as well as psychometrics and instrument validity.

We suggest the possible inclusion of the authors previously mentioned in the methodological appraisal. It is also important to review the references, as references numbers 4, 7, 20, and 21 appear to be incomplete.

Summary

This is a high-quality article suitable for publication in several types of journals within the fields of public health, primary health care, mental health, addictions, and instrument validation. The conceptual and methodological foundation is very solid and scientifically relevant.

The manuscript could be improved in terms of rigour and detail through minor revisions concerning the concepts of face validity and content validity, clarification of the cognitive interview procedures, reinforcement of the idea that the study does not merely involve the adaptive translation of a measurement instrument, greater emphasis on the importance of culturally embedded alcohol consumption, possible enhancement of Table 5 (with explicit justification for the adaptations), and, above all, the formal inclusion of a section on study limitations.

**********

what does this mean?). If published, this will include your full peer review and any attached files.

If you choose “no”, your identity will remain anonymous but your review may still be made public.

**Do you want your identity to be public for this peer review?**  For information about this choice, including consent withdrawal, please see our Privacy Policy

Reviewer #1: **Yes:**  Raul Cordeiro

Reviewer #2: **Yes:**  Luís de Oliveira Nabais

**********

[NOTE: If reviewer comments were submitted as an attachment file, they will be attached to this email and accessible via the submission site. Please log into your account, locate the manuscript record, and check for the action link "View Attachments". If this link does not appear, there are no attachment files.]

To ensure your figures meet our technical requirements, please review our figure guidelines: https://journals.plos.org/plosone/s/figures

You may also use PLOS’s free figure tool, NAAS, to help you prepare publication quality figures: https://journals.plos.org/plosone/s/figures#loc-tools-for-figure-preparation.

NAAS will assess whether your figures meet our technical requirements by comparing each figure against our figure specifications.

# Author comment: Author response to Decision Letter 1

**DOI:** 10.1371/journal.pone.0359625.r002

28 Jul 2026

We thank the Academic Editor and reviewers for their constructive comments and suggestions. We have carefully addressed each comment in the attached "Response to Reviewers" document, and all corresponding revisions have been incorporated into the revised manuscript.

Attachment Submitted filename: Response to Reviewers.docx

# Peer review: Decision Letter 1

**DOI:** 10.1371/journal.pone.0359625.r003

**Authors:** Maria Nogueira

16 Sep 2026

Strengthening alcohol consumption screening in primary care for culturally diverse populations in Latin America: adaptation and content and face validity of the AUDIT in Guaviare, Colombia

PONE-D-25-64964R1

Dear Dr. Andrea López-González

We’re pleased to inform you that your manuscript has been judged scientifically suitable for publication and will be formally accepted for publication once it meets all outstanding technical requirements.

Within one week, you’ll receive an e-mail detailing the required amendments. When these have been addressed, you’ll receive a formal acceptance letter and your manuscript will be scheduled for publication.

An invoice will be generated when your article is formally accepted. Please note, if your institution has a publishing partnership with PLOS and your article meets the relevant criteria, all or part of your publication costs will be covered. Please make sure your user information is up-to-date by logging into Editorial Manager at Editorial Manager® and clicking the ‘Update My Information' link at the top of the page. For questions related to billing, please contact billing support.

If your institution or institutions have a press office, please notify them about your upcoming paper to help maximize its impact. If they’ll be preparing press materials, please inform our press team as soon as possible -- no later than 48 hours after receiving the formal acceptance. Your manuscript will remain under strict press embargo until 2 pm Eastern Time on the date of publication. For more information, please contact onepress@plos.org.

Kind regards,

Maria José Nogueira, Ph.D.

Academic Editor

PLOS One

Additional Editor Comments (optional):

The authors considered the main issues raised and introduced revisions that clearly strengthened the conceptual, methodological, and scientific consistency of the article.

The current version of the manuscript can be accepted for publication.

Reviewers' comments:

Reviewer's Responses to Questions

**Comments to the Author**

Reviewer #1: All comments have been addressed

Reviewer #2: All comments have been addressed

**********

2. Is the manuscript technically sound, and do the data support the conclusions?

Reviewer #1: Yes

Reviewer #2: Yes

**********

3. Has the statistical analysis been performed appropriately and rigorously?

Reviewer #1: Yes

Reviewer #2: Yes

**********

4. Have the authors made all data underlying the findings in their manuscript fully available?

The PLOS Data policy

Reviewer #1: Yes

Reviewer #2: Yes

**********

5. Is the manuscript presented in an intelligible fashion and written in standard English?

Reviewer #1: Yes

Reviewer #2: Yes

**********

Reviewer #1: (No Response)

Reviewer #2: The revised version of the manuscript provides an appropriate response to the suggestions and recommendations made in the previous review. Overall, the authors have taken into consideration the main issues raised and have introduced revisions that have clearly strengthened the conceptual, methodological, and scientific consistency of the article.

In particular, the distinction between translation and cultural adaptation has been further clarified, making the specific nature of the study more explicit and reinforcing its originality. The suggestions concerning the concepts of face validity and content validity have also been addressed, and the procedures associated with the cognitive interviews have been described in greater detail, thereby contributing to greater methodological rigor.

The revisions made to the presentation of the results, particularly in Table 5, provide a clearer understanding of the rationale underlying the adaptations and more clearly demonstrate that the process undertaken went beyond a mere linguistic adaptation of the instrument. The greater emphasis placed in the Discussion and Conclusions on the issue of “guarapo” and the cultural invisibility of certain forms of alcohol consumption is also particularly relevant, as this represents one of the most significant and innovative findings of the study.

Of particular importance is the inclusion of a specific section addressing the study’s limitations. This directly responds to one of the main recommendations made in the previous review and provides a more appropriate framework for understanding the methodological choices and the scope of the findings.

The suggestions concerning the strengthening of the bibliographic background have also been taken into consideration, and the references have been reviewed accordingly, contributing to a more robust theoretical and methodological framework.

Overall, the revisions satisfactorily address the issues raised in the previous review and substantially improve the quality and coherence of the manuscript. The present version is more consistent, both in its account of the cultural adaptation process and in the articulation between the methodology, results, and discussion.

In view of the revisions introduced and the overall quality of the revised manuscript, I consider that the manuscript has been significantly improved and is suitable for publication in its present form.

**********

what does this mean?). If published, this will include your full peer review and any attached files.

If you choose “no”, your identity will remain anonymous but your review may still be made public.

**Do you want your identity to be public for this peer review?**  For information about this choice, including consent withdrawal, please see our Privacy Policy

Reviewer #1: **Yes:**  Raul Cordeiro

Reviewer #2: **Yes:**  Luís de Oliveira Nabais

**********

# Editor report: Acceptance letter

**DOI:** 10.1371/journal.pone.0359625.r004

**Authors:** Maria Nogueira

PONE-D-25-64964R1

PLOS One

Dear Dr. López-González,

I'm pleased to inform you that your manuscript has been deemed suitable for publication in PLOS One. Congratulations! Your manuscript is now being handed over to our production team.

At this stage, our production department will prepare your paper for publication. This includes ensuring the following:

* All references, tables, and figures are properly cited

* All relevant supporting information is included in the manuscript submission,

* There are no issues that prevent the paper from being properly typeset

You will receive further instructions from the production team, including instructions on how to review your proof when it is ready. Please keep in mind that we are working through a large volume of accepted articles, so please give us a few days to review your paper and let you know the next and final steps.

Lastly, if your institution or institutions have a press office, please let them know about your upcoming paper now to help maximize its impact. If they'll be preparing press materials, please inform our press team within the next 48 hours. Your manuscript will remain under strict press embargo until 2 pm Eastern Time on the date of publication. For more information, please contact onepress@plos.org.

You will receive an invoice from PLOS for your publication fee after your manuscript has reached the completed accept phase. If you receive an email requesting payment before acceptance or for any other service, this may be a phishing scheme. Learn how to identify phishing emails and protect your accounts at https://explore.plos.org/phishing.

If we can help with anything else, please email us at customercare@plos.org.

Thank you for submitting your work to PLOS One and supporting open access.

Kind regards,

PLOS One Editorial Office Staff

on behalf of

Professor Maria José Nogueira

Academic Editor

PLOS One
